# A Comparative Study on the Thermodynamics of Halogen Bonding of Group 10 Pincer Fluoride Complexes

**DOI:** 10.1002/chem.201904863

**Published:** 2020-02-28

**Authors:** Markus Joksch, Hemlata Agarwala, Monica Ferro, Dirk Michalik, Anke Spannenberg, Torsten Beweries

**Affiliations:** ^1^ Leibniz-Institut für Katalyse e.V. an der Universität Rostock Albert-Einstein-Strasse 29a 18059 Rostock Germany; ^2^ Department of Synthetic Molecular Chemistry Ångström Laboratory, Box 523 Uppsala University Lägerhyddsvägen 1 75120 Uppsala Sweden; ^3^ Politecnico di Milano Dipartimento di Chimica Materiali e Ing. Chimica “G. Natta” Via L. Mancinelli 7 20131 Milano Italy; ^4^ Institut für Chemie Universität Rostock Albert-Einstein-Strasse 3a 18059 Rostock Germany

**Keywords:** halogen bonding, nickel, palladium, pincer complexes, platinum

## Abstract

The thermodynamics of halogen bonding of a series of isostructural Group 10 metal pincer fluoride complexes of the type [(3,5‐R_2_‐^*t*Bu^POCOP^*t*Bu^)MF] (3,5‐R_2_‐^*t*Bu^POCOP^*t*Bu^=*κ* ^3^‐C_6_HR_2_‐2,6‐(OP*t*Bu_2_)_2_ with R=H, *t*Bu, COOMe; M=Ni, Pd, Pt) and iodopentafluorobenzene was investigated. Based on NMR experiments at different temperatures, all complexes **1‐*t*Bu** (R=*t*Bu, M=Ni), **2‐H** (R=H, M=Pd), **2‐*t*Bu** (R=*t*Bu, M=Pd), **2‐COOMe** (R=COOMe, M=Pd) and **3‐*t*Bu** (R=*t*Bu, M=Pt) form strong halogen bonds with Pd complexes showing significantly stronger binding to iodopentafluorobenzene. Structural and computational analysis of a model adduct of complex **2‐*t*Bu** with 1,4‐diiodotetrafluorobenzene as well as of structures of iodopentafluorobenzene in toluene solution shows that formation of a type I contact occurs.

## Graphical abstract

**Pincer complexes studied**: Group 10 POCOP (*κ* ^3^‐C_6_HR_2_‐2,6‐(OP*t*Bu_2_)_2_; R=H, *t*Bu) fluoride complexes show pronounced halogen bonding with iodopentafluorobenzene in toluene. Along an isostructural series of complexes, Pd fluorides show the strongest interactions.

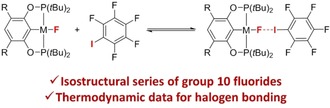

## Introduction

Halogen bonding (XB), a non‐covalent interaction between electron‐deficient halogen atoms and a Lewis‐basic site, has attracted considerable attention in recent years due to its relevance for numerous applications including functional materials, supramolecular polymers, molecular recognition, anion receptors, crystal engineering, and organocatalysis.^1^, ^2^ Halogen bonding is a highly directional interaction that can be described using different models, one of them being the so‐called σ hole that results from an anisotropic electron distribution around halogen atoms when being covalently bound to a residue R. This effect is most pronounced for electron‐withdrawing moieties R and results in a highly positive region in elongation of the covalent R−X bond that is capable of acting as an electron‐accepting site.^3^

**Figure 1:**
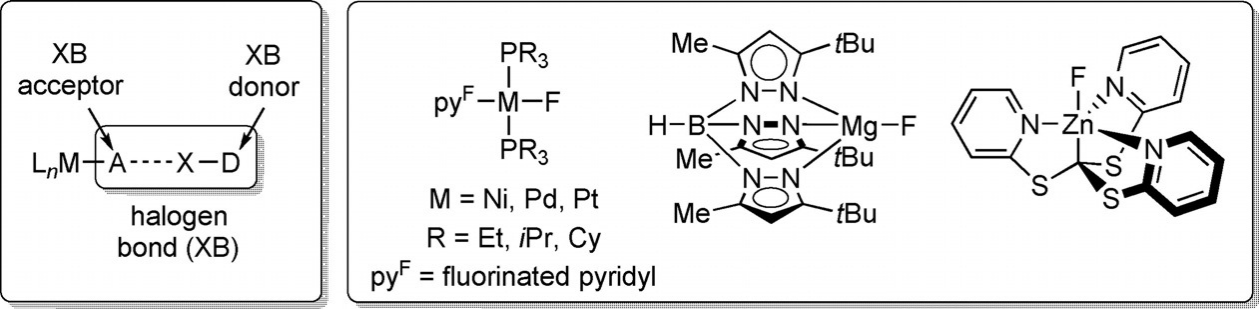
Schematic depiction of halogen bonding of transition‐metal halide complexes L_*n*_M−A, and metal fluoride complexes used to date for characterization of XB in solution.

Numerous studies of halogen bonding of ubiquitous organo‐halogen compounds in solution and in the solid state have been reported and demonstrate the enormous potential of this type of interaction.^4^ However, similar investigations involving analogous inorganic or organometallic structures such as metal halides or halide complexes are still comparably rare (Figure 1). This is somewhat surprising given that transition‐metal complexes containing halide ligands are known to act as precatalysts and even as active species in a large number of catalytic processes.^5^ Halogen‐bonding interactions could, in principle, be of relevance for C−X bond activation reactions as it was shown by Huber et al. for organocatalytic reactions.^6^

**Scheme 1:**
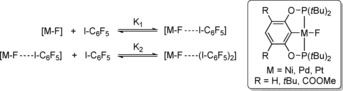
Schematic depiction of halogen bonding of late transition‐metal halide complexes with C_6_F_5_I and structural motif of pincer complexes used in this study.

Seminal structural investigations by Brammer and co‐workers suggest that transition‐metal–halide complexes are excellent hydrogen‐ and halogen‐bonding acceptors.^7^ More recent work by others further supported this and broadened the scope of organometallic halogen bonding.^8^ However, systematic studies of halogen bonding in solution to access thermodynamic data of these interactions are still underrepresented, yet highly desirable in order to fully understand all aspects of this phenomenon and further promote this field of research in terms of applicability. Metal fluorides are ideal for these studies because the ^19^F NMR resonance of the fluoride ligand in most cases is the only ^19^F NMR signal and/or appears isolated from other signals. Moreover, it is highly sensitive towards changes in its electronic environment. The formation of XB adducts can thus be readily monitored by NMR spectroscopy and the thermodynamics of the interactions can be quantified using the ^19^F NMR shift of the metal fluoride. Parkin and co‐workers reported halogen bonding of magnesium^9^ and zinc^10^ fluoride complexes (Figure 1) with iodopentafluorobenzene, C_6_F_5_I, which was found to be an excellent XB donor due to the presence of a perfluorinated aryl ring and the resulting high Lewis acidity of the iodine donor atom. A more systematic study of halogen bonding of late‐transition‐metal fluorides was presented by Perutz and Brammer^11^ who used a series of Group 10 bis(phosphine) complexes that form monofluorides upon C−F activation of fluorinated aromatics such as hexafluorobenzene and fluorinated pyridines (Figure 1). Upon interaction of these fluoride complexes with the XB donor C_6_F_5_I significant shifts of the metal fluoride ^19^F NMR resonance of up to 30 ppm were observed. Thermodynamic data reported for these interactions suggest the formation of strong halogen bonds for all complexes (2.4<*K*_300_<5.2; −26<Δ*H* ^o^<−16 kJ mol^−1^; −73<Δ*S* ^o^<−42 J K^−1^ mol^−1^). Also, depending on the polarity of the solvent, changes in the stoichiometry of adduct formation were observed (Figure 1). Recently, also perfluoroalkyl iodide and perfluoroaryl bromide donors have been evaluated with related Ni bis(phosphine) systems.^12^ However, in these studies no full isostructural series of complexes was investigated as depending on the metal centre and the phosphine ligand different patterns of C−F activation were observed, thus resulting in different binding modes of the fluorinated pyridine ligand.^13^ It should be noted that in previous studies, attempts to prepare an isostructural series of monofluoride complexes failed and to the best of our knowledge, no such series of complexes is known to date. The necessity of using monofluoride complexes arises from the problem of formation of multiple interactions (Scheme 1) in the case of well‐precedented di‐ or oligofluoride species such as Group 4 metallocene difluorides or complex ions such as [MF_6_]^*n*−^.

**Scheme 2:**
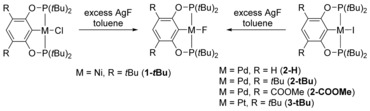
Synthesis of fluoride complexes.

Very recently, we have studied halogen bonding of a series of isostructural Group 10 pincer iodide complexes of the type [(3,5‐R_2_‐^*t*Bu^POCOP^*t*Bu^)MI] (3,5‐R_2_‐^*t*Bu^POCOP^*t*Bu^=*κ* ^3^‐C_6_HR_2_‐2,6‐(OP*t*Bu_2_)_2_ with R=*t*Bu; M=Ni, Pd, Pt) with iodine and found that these complexes form strong halogen bonds in the solid state.^14^ Also, the pincer ligand represents an excellent platform to access a large number of isostructural complexes (Scheme 2).^15^ Based on these findings, we now present a comparative study of halogen bonding of isostructural Group 10 fluoride complexes with C_6_F_5_I in solution.

## Results and Discussion

Synthesis of nickel, palladium and platinum POCOP fluoride complexes was accomplished by salt metathesis reactions with AgF in toluene starting from suitable halide precursors (Scheme 2).

For these reactions it is highly desirable to strictly exclude the presence of water because the formed fluoride ligand is highly nucleophilic and thus readily forms hydrogen bonding adducts with water. Also, formation of bifluoride complexes, L_*n*_M‐FHF, is a major problem, which must be addressed by rigorous drying of solvents and removing traces of HF from AgF. In cases in which traces of the metal bifluoride were present after fluorination, these were removed by treating the crude material with a NaH suspension, followed by filtration to yield the pure metal fluoride complexes. Nickel fluoride complex **1‐*t*Bu** was obtained from the corresponding chloride complex as a yellow solid in 58 % yield. In the case of palladium and platinum complexes **2‐H** ^15b^, **2‐*t*Bu**, **2‐COOMe** and **3‐*t*Bu**, salt metathesis from the iodide species was found to be more convenient, giving the desired fluorides as colourless microcrystalline solids in yields of up to 69 %.

All complexes were assigned as metal fluorides based on isolated ^19^F NMR resonances in the highfield region of the NMR spectra (Table 1). These values resemble those found for other late‐transition‐metal fluorides.^16^ ^31^P NMR data are well in line with those reported for structurally similar square‐planar Group 10 pincer halide complexes that possess P(*t*Bu)_2_ donor sites.^17^ It should be noted that trends observed for the ^31^P NMR resonances of complexes described herein were observed before for iodide complexes bearing these ligands.^15^ In all cases ^31^P NMR shifts of the metal fluoride complex were found highfield from values found for isostructural iodide and chloride complexes, a trend that was observed earlier by Campora and co‐workers for PCP Ni and Pd halide complexes.^18^ Notably, P−F coupling was only observed for **1‐*t*Bu** and **3‐*t*Bu** (Table 1) and ^31^P NMR resonances for Pd complexes were comparably broad. Small values for ^2^ *J*_P,F_ or no detectable coupling^14^ at room temperature was observed before for related Group 10 pincer fluoride complexes (e.g. [(PCP)PdF] (PCP=*κ* ^3^‐C_6_H_3_‐2,6‐(CH_2_P*i*Pr_2_)_2_), ^2^ *J*_P,F_=5.9 Hz).^18^ Prior to titration experiments, the absence of hydrogen bound H_2_O or HF was confirmed by ^1^H NMR analysis (absence of additional resonances in the downfield region, that is, up to *δ*=15 ppm) and by CHN analysis, which showed good agreement with expected values.

**Table 1:** Selected NMR data of complexes **1‐*t*Bu**, **2‐H**, **2‐*t*Bu**, **2‐COOMe**, and **3‐*t*Bu** (*δ* in ppm, 297 K, [D_8_]toluene).

	^19^F NMR	^31^P NMR
**1‐*t*Bu**	−380.1 (t, *J*_P,F_=27.0 Hz)	179.1 (d, *J*_P,F_=27.0 Hz)
**2‐H**	−317.8 (s)	185.8 (s)
**2‐*t*Bu**	−315.6 (s)	185.6 (s)
**2‐COOMe**	−326.3 (s)	191.0 (s)
**3‐*t*Bu**	−309.9 (s); −309.9 (d, *J*_Pt,F_=136.0 Hz)	174.3 (d, *J*_P,F_=2.0 Hz); 174.3 (dd, *J*_Pt,P_=3215 Hz, *J*_P,F_=2.0 Hz)

**Figure 2:**
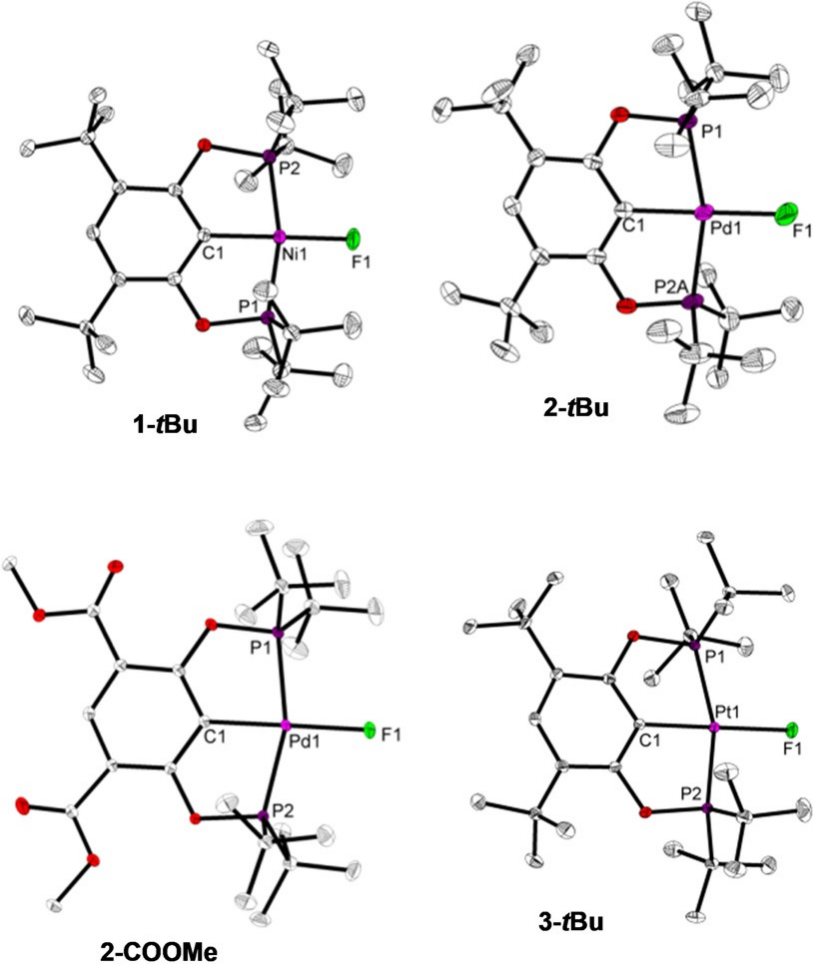
Molecular structures of metal fluoride complexes. Thermal ellipsoids correspond to 30 % probability. Hydrogen atoms are omitted for clarity. For complex **1‐*t*Bu**, the second molecule of the asymmetric unit is not shown. For complex **2‐*t*Bu**, minor occupancy atoms of the disordered POCOP ligand are removed for clarity.

The molecular structure of complex **1‐*t*Bu** was confirmed by single crystal X‐ray diffraction (SC‐XRD, Figure 2). The asymmetric unit contains two molecules of the complex in distorted square‐planar coordination geometry with a terminal fluoride ligand (Ni1−F1 1.8417(13), Ni2−F2 1.8418(12) Å). These values are in the same range as those found before for other Ni PCP pincer fluoride complexes.^18^, ^19^ In the space‐fill model (Figure S6, Supporting Information) it can be seen that the sterically demanding *t*Bu groups shield the fluoride ligand, which could be of importance for selective formation of a 1:1 XB adduct. Additionally, we analysed the molecular structure of the isostructural complexes **2‐*t*Bu**, **2‐COOMe** and **3‐*t*Bu** (Figure 2). Bond lengths and angles of the Pd and Pt complexes are in the expected range with the M−F bonds (**2‐*t*Bu**: 2.0304(16), **2‐COOMe**: 2.0317(12), **3‐*t*Bu**: 2.0539(15) Å) being significantly longer than in the Ni complex **1‐*t*Bu** due to the larger size of the heavier congeners.

Fluoride complexes of this type being prone to coordination of Lewis bases can be seen from the interaction of the Pd−F moiety with H_2_O in complex **2‐*t*Bu⋅H_2_O**, which is formed in the presence of trace amounts of H_2_O (Figure S7, Supporting Information).^20^ Notably, formation of such adducts was only observed in case of Pd, indicating a higher tendency of these complexes to perform such interactions. Spectroscopic XB studies thus have to be carried out under rigorous exclusion of moisture.

**Figure 3:**
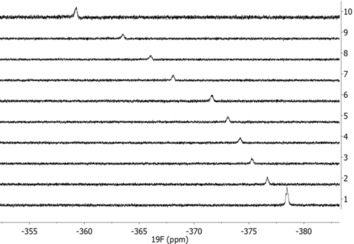
Stacked plot of ^19^F NMR spectra (toluene, 242.3 K) at different molar ratios of [C_6_F_5_I]**1‐*t*Bu**, showing the shift to lower field of the nickel fluoride resonance. Spectra correspond to the following ratios C_6_F_5_I**1‐*t*Bu**: 1: 0; 2: 0.51; 3: 1.10; 4: 1.57; 5: 2.17; 6: 3.14; 7: 4.94; 8: 7.05; 9: 10.23; 10: 19.81.

NMR titrations were carried out in toluene using complexes shown in Scheme 1 as the XB acceptor and C_6_F_5_I as the XB donor, which was used before in related studies. When adding the halogen‐bond donor, the ^19^F NMR resonance of the fluoride ligand of all complexes exhibits a significant shift to lower field (Figure 3).

The chemical shift difference Δ*δ* of pure metal fluoride and its C_6_F_5_I adduct (formed in the presence of an excess of C_6_F_5_I) did not change significantly when going from lowest to highest temperature for all complexes. Although Δ*δ* is similar for Ni and Pd complexes, **3‐*t*Bu** showed much smaller lowfield shifts during titration.

Minor variations of the ^19^F NMR chemical shifts of C_6_F_5_I are due to the changing concentration and can be neglected. Also, ^31^P NMR signals for the phosphine ligands show no significant changes. This indicates that the interaction takes place exclusively between metal fluoride and the iodine of C_6_F_5_I and ^19^F NMR spectroscopy is thus well suited for a determination of the strength of this contact.

**Figure 4:**
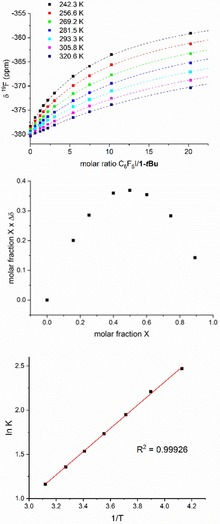
Top: Titration data at different temperatures, showing observed values for the ^19^F NMR chemical shift of the metal fluoride *δ* ^19^F versus molar ratio of C_6_F_5_I and complex **1‐*t*Bu**. Centre: Job plot for XB of complex **1** and C_6_F_5_I (toluene, [**1‐*t*Bu**]+[C_6_F_5_I]=0.01 mol L^−1^). Bottom: van't Hoff plot for XB of C_6_F_5_I and complex **1‐*t*Bu**.

The resulting titration curves recorded at different temperatures using metal fluoride and C_6_F_5_I stock solutions of similar concentrations (Figure 4) was fitted using a simple model of 1:1 binding. All curves show a reasonable amount of data points before saturation occurs, indicating that the concentration regime used for titration experiments is sensible. From this fit, equilibrium constants *K* for XB adduct formation as well as the value *δ*_max_, which corresponds to the chemical shift of the XB adduct of 1:1 stoichiometry, were obtained. Notably, analysis of the data using 1:2 stoichiometry gave much poorer results with fitted data clearly deviating from the experimental values for all systems investigated.

Formation of adducts of 1:1 stoichiometry was exemplarily confirmed by Job plots for complex **1‐*t*Bu** and C_6_F_5_I. For the Job plot, changes in the chemical shift of the ^19^F NMR resonance (Δ*δ*) were monitored as a function of the molar fraction of metal complex (X) while keeping the sum of the concentrations of **1‐*t*Bu** and C_6_F_5_I constant at a value of 0.01 mol L^−1^. As expected for a 1:1 complex, the graph of XΔ*δ* versus *X* shows a maximum at *X*=0.5 (Figure 4). Analysis of the temperature dependence of *K* (van't Hoff plot, Figure 4) gives values for enthalpy Δ*H* ^o^ and entropy Δ*S* ^o^. An overview of thermodynamic data is depicted in Table 2.

**Table 2:** Thermodynamic data for XB of Group 10 pincer fluoride complexes in toluene (errors are 95 % confidence limits and are based on statistics of fits).

	*K*_242.3_	*K*_305.8_	Δ*H* ^o^ [kJ mol^−1^]	Δ*S* ^o^ [J K^−1^ mol^−1^]	Δ*δ*_max, 305.8_ [ppm]^[b]^
**1‐*t*Bu**	11.8(1)	3.88(4)	−10.9(5)	−24(2)	33.0
**2‐H**	93(1)^[a]^	36.5(4)	−13.7(2)	−15(1)	33.5
**2‐t*B*u**	116(1)	33.1(4)	−12.1(4)	−10(1)	32.2
**2‐COOMe**	105(2)^[a]^	22.2(2)	−18.6(7)	−34(3)	32.1
**3‐*t*Bu**	24.1(2)	8.9(1)	−9.2(3)	−12(1)	21.7

[a] At 256.6 K (due to limited solubility of complexes **2‐H** and **2‐COOMe**). [b] The chemical shift difference Δ*δ*_max,305.8_ between free metal fluoride and metal fluoride−C_6_F_5_I adduct at 305.8 K was calculated by the fitting routine.

Perutz et al. described variations of the stoichiometry of adduct formation when using less polar solvents such as heptane11a that do not allow for additional interactions, for example, π–π interactions between toluene and C_6_F_5_I. In our case, use of nonpolar solvents was not possible because all complexes show very limited solubility. In fact, solubility of **2‐H** in toluene is rather poor at low temperatures, NMR titrations using this complex were only done at temperatures between 268 and 313 K.

Complexes **1‐*t*Bu**, **2‐*t*Bu**, and **3‐*t*Bu** are isostructural and differ only in the metal centre and are thus comparable. The results show similar binding enthalpies −Δ*H* ^o^ with almost identical values for **1‐*t*Bu** and **2‐*t*Bu**, whereas −Δ*H* ^o^ for the Pt analogue **3‐*t*Bu** is significantly smaller. This is in contrast to results presented earlier by Perutz et al. for structurally similar, but not isostructural complexes, where −Δ*H* ^o^ was observed to follow the trend Ni<Pd<Pt.11b Also, a correlation of −Δ*H* ^o^ with −Δ*S* ^o^ was observed leading to compensation in the values of Δ*G* ^o^ and *K*. Especially in the case of the Pd complex **2‐*t*Bu**, this is not possible because the value determined for −Δ*S* ^o^ is much smaller than for the Ni complex **1‐*t*Bu** and in the same range as for the Pt complex **3‐*t*Bu**. It should be noted that binding constants are much larger for Pd complexes than for Ni and Pt complexes (Table 2). Also, despite showing excellent agreement with 1:1 binding, titration curves for Pd complexes appeared slightly different: although at lower ratios of [C_6_F_5_I][Pd] a large variation of chemical shift differences Δ*δ*_Τ_ was observed with temperature, this difference decreases when going to higher ratios [C_6_F_5_I][Pd]. For Ni and Pt, Δ*δ*_Τ_ continuously increases, giving maximum values at high concentrations of the donor. A similar behaviour was found before for the Pd complex [Pd(F)(4‐C_5_NF_4_)(PCy_3_)_2_], pointing towards the presence of subtle differences in XB systems containing Pd complexes. Stronger binding in case of the Pd complexes is also evidenced by a qualitative comparison of Job plots for complexes **1‐*t*Bu** and **2‐COOMe**, in which the curve for **2‐COOMe** showed a much larger amplitude with close to triangular shape (Figures S13 and S14, Supporting Information).^21^

The difference in values for −Δ*H* ^o^ and −Δ*S* ^o^ for compounds **2‐H**, **2‐*t*Bu** and **2‐COOMe**, showing variations of the substitution pattern of the POCOP ligand is significant, suggesting that the electronic nature of the ligand plays a role for the strength of the XB interaction. However, the observed trend in −Δ*H* ^o^ **2‐H**<**2‐*t*Bu**<**2‐COOMe** is somewhat counterintuitive, giving largest values for the complex possessing an electron withdrawing COOMe substituted POCOP ligand and smallest values for the more electron‐rich *t*Bu substituted complex. XB occurs by interaction of the partially positive XB donor with a Lewis‐basic fluoride atom and should thus be most pronounced for complex **2‐*t*Bu**. The rationalization of the herein observed effect is currently under investigation.

**Figure 5:**
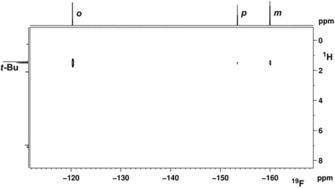
^19^F,^1^H HOESY spectrum of **1‐*t*Bu**/C_6_F_5_I (470.5/500.1 MHz in [D_8_]toluene, mixing time 1.7 s).

To further support the formation of an XB adduct in solution, we performed ^19^F,^1^H HOESY NMR experiments for the system **1‐*t*Bu**C_6_F_5_I using a tenfold excess of the XB donor. The ^19^F,^1^H Heteronuclear Overhauser Effect SpectroscopY (HOESY) NMR spectrum reveals the presence of intermolecular contacts between the *t*Bu protons of the metal fluoride and the C_6_F_5_I fluorine nuclei. The heteronuclear NOE correlation with the CH_3_/*o*‐F is significantly more intense than the ones for CH_3_/*m*‐F and CH_3_/*p*‐F (Figure 5). This indicates that the iodine is interacting with the metal fluoride, but in the XB adduct the *p*‐F is remote from the *t*Bu group and only produces a small NOE. Similar effects were observed before in a XB study of 1,4‐diazabicyclo[2.2.2]octane and C_6_F_5_I.^22^

**Figure 6:**
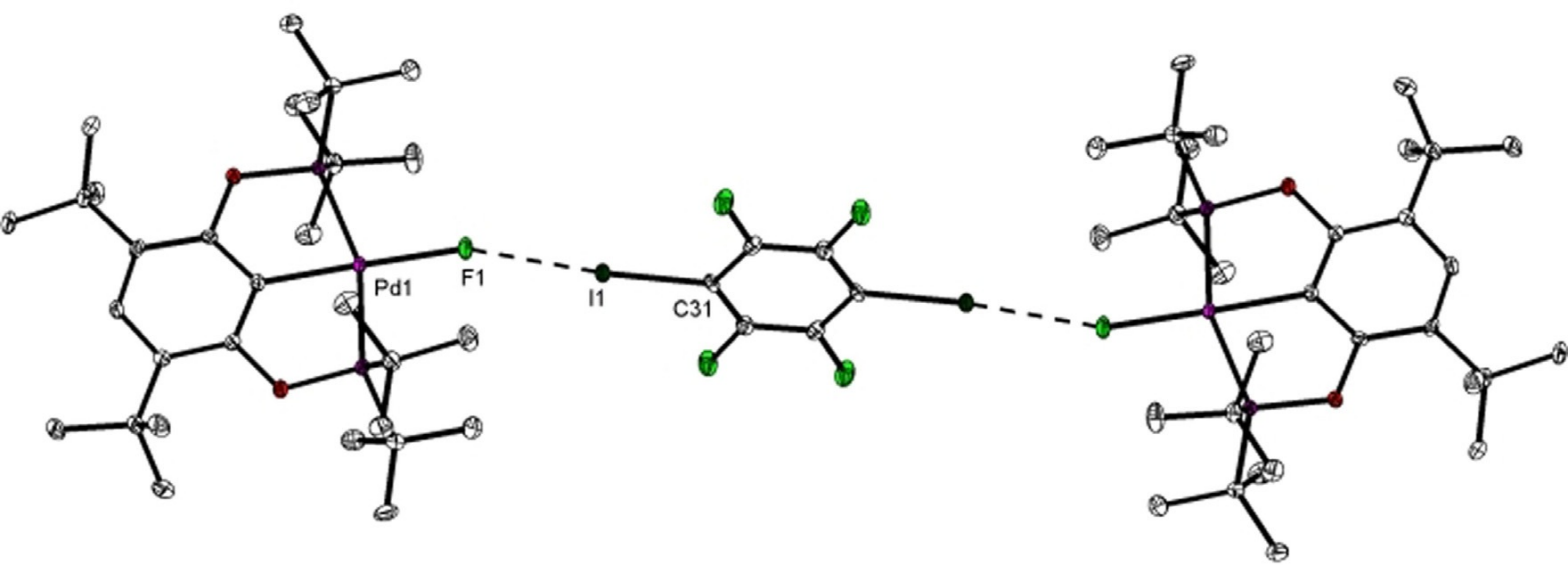
Molecular structure of compound **2‐*t*Bu⋅I‐C_6_F_4_‐I**. Thermal ellipsoids correspond to 30 % probability. Hydrogen atoms are omitted for clarity.

Adducts formed between metal fluorides and C_6_F_5_I in toluene solution could unfortunately not be crystallized. To get additional structural insights into halogen bonding of these pincer systems, we co‐crystallized complex **2‐*t*Bu** with 1,4‐diiodotetrafluorobenzene, C_6_F_4_I_2_ and used this as a benchmark for further computational studies of solution structures formed with C_6_F_5_I (see above). C_6_F_4_I_2_ was used before as a donor for structural studies of halogen bonding of palladium pincer complexes.^23^ Co‐crystallization of **2‐*t*Bu** and C_6_F_4_I_2_ in 2:1 ratio from toluene yields the adduct **2‐*t*Bu⋅I‐C_6_F_4_‐I**. The molecular structure is shown in Figure 6. It shows two molecules of **2‐*t*Bu** bridged by one molecule of the bifunctional XB donor. Notably, both palladium fluoride XB acceptors are in coplanar arrangement, whereas the bridging C_6_F_4_I_2_ is tilted out of this plane by 78°.

Given that the orientation of the molecules with respect to each other in a crystal might arise due to various packing forces in the crystal, especially in halogen bonded systems, density functional theory (DFT) calculations were carried out for **2‐*t*Bu⋅I‐C_6_F_4_‐I**, using the crystal‐structure coordinates for the input geometry. It was indeed found that the two molecules of **2‐*t*Bu** are not co‐planar in the lowest‐energy optimized structure in toluene. They are at 79.21° with respect to each other (Figure S15 a, Supporting Information). Furthermore, the angle between the plane passing through I−C_6_F_4_−I and the planes through each of the two **2‐*t*Bu** molecules are 23.36° and 56.15° (Figures S15 b, S15 c).

A comparison of the Pd1−F1 distance in the adduct structure (2.0681(17) (SC‐XRD); 2.0847 Å (DFT); Table S9 a, Supporting Information) with that of the free acceptor **2‐*t*Bu** (2.0304(16) (SC‐XRD); 2.0670 Å (DFT); Table S10 a) shows that upon interaction an elongation of the metal–fluorine bond by approximately 2 % occurs. The F⋅⋅⋅I halogen bonding contact is characterized by a F1⋅⋅⋅I1 distance of 2.6828(18) Å (DFT: 2.7020 Å; Table S9 a), which is clearly lower than the sum of the van der Waals radii (svdW=3.45 Å). Along with this, the bond C31−I1 is slightly elongated (2.115(3) (SC‐XRD), 2.1231 Å (DFT); Table S9 a) compared with the value found for the free XB donor (2.079(4),^24^ Σ*r*_cov_=2.08 Å).^25^ Analysis of the bond angles along the XB interaction shows a slightly bent arrangement (SC‐XRD: Pd1‐F1‐I1: 160.0°, DFT: 166.3°; SC‐XRD: F1‐I1‐C31: 174.4°, DFT: 178.4°; Table S9 a). In a previous structural study Whitwood, Brammer, and Perutz showed that the degree of bending of the M−X⋅⋅⋅I−C unit is strongly dependent on the halide X of the acceptor component with fluorides resulting in linear arrangements whereas structures of metal iodide adducts are bent significantly.8e Notably, XB interactions of electron deficient σ holes with regions of higher electron density are typically strongly bent. Reasons for the herein found almost linear arrangement can be the steric hindrance due to the presence of *t*Bu groups, the more isotropic electron distribution of the fluoride ligand, or simple packing requirements. In the solid state the shortest distance between protons of the P(*t*Bu)_2_ group and *o*‐F atoms of C_6_F_4_I_2_ is found for F2⋅⋅⋅H18B (4.634 Å). This corresponds to the most pronounced HOESY correlation in related systems with C_6_F_5_I in solution (Figure 5).

In a previous structural study Rissanen and Wendt showed that co‐crystallisation of structurally similar PCP Pd chloride and bromide complexes (PCP=2,6‐bis[(di‐*tert*‐butylphosphino)methyl]phenyl) with C_6_F_4_I_2_ results in the formation of adducts with multiple binding of the XB donor to the Pd halide moiety. Despite similarities in the metal halide environments we did not observe this binding mode in our case because the fluoride‐acceptor component displays a less anisotropic electron distribution than chloride or bromide.^26^

**Figure 7:**
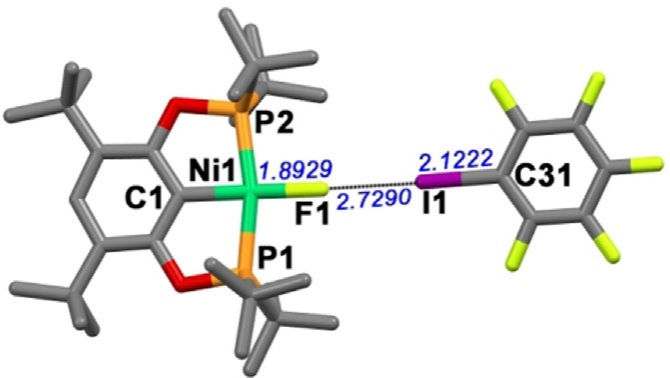
Optimized structure for the adduct **1‐*t*Bu⋅C_6_F_5_I**, formed between complex **1** and C_6_F_5_I in toluene. Distances are in Å.

To further gain a structural understanding of the halogen bonded adducts of isostructural complexes **1‐*t*Bu**, **2‐*t*Bu**, and **3‐*t*Bu** with C_6_F_5_I, we obtained the minimum energy DFT‐optimized structures of the adducts by making minor modifications in the crystal structure of **2‐*t*Bu⋅I‐C_6_F_4_‐I**. The optimizations were carried out using four different DFT functionals: B3PW91, BHandHLYP, M06, and B3LYP. For the free acceptors, the functionals producing the minimum‐energy optimized structure closest to the crystal structure were BHandHLYP (for **1‐*t*Bu**; see root mean square error (RMSE) values in Tables S11 a and S11b, Supporting Information) and M06 (for **2‐*t*Bu** and **3‐*t*Bu**; see RMSE values in Tables S10 a, S10 b, S12 a and S12 b). Nevertheless, B3LYP appears to give the closest resemblance to the real situation when it comes to halogen‐bonded interactions in the present set of complexes as can be noted in the bond lengths and angles for **2‐*t*Bu⋅I‐C_6_F_4_‐I** (see RMSE values in Tables S9 a and S9 b). Therefore, the structures of the adducts with C_6_F_5_I obtained using B3LYP functional in toluene will be considered in the following discussion. As an example, the structure of the adduct of complex **1‐*t*Bu** and C_6_F_5_I is depicted in Figure 7. The bond lengths and angles for the adducts **X⋅C_6_F_5_I** (**X**=**1‐*t*Bu**, **2‐*t*Bu**, **3‐*t*Bu**) are listed in Tables S12–S14. The F⋅⋅⋅I halogen bonding contact is characterized by F1⋅⋅⋅I1 distances of 2.7290 (**1‐*t*Bu⋅C_6_F_5_I**), 2.6628 (**2‐*t*Bu⋅C_6_F_5_I**), and 2.6828 Å (**3‐*t*Bu⋅C_6_F_5_I**), which are lower than the sum of the van der Waals radii (svdW 3.45 Å). Also, the bond C31−I1 is slightly elongated (2.1222 (**1‐*t*Bu⋅C_6_F_5_I**), 2.1283 (**2‐*t*Bu⋅C_6_F_5_I**), and 2.1254 Å (**3‐*t*Bu⋅C_6_F_5_I**)) in comparison with that in the free XB donor (2.079(4),^24^ Σr_cov_=2.08 Å).^25^ The bond angles along the XB interaction reveal an almost linear arrangement: M1‐F1‐I1 (M=metal): 177.36° (**1‐*t*Bu⋅C_6_F_5_I**), 160.30° (**2‐*t*Bu⋅C_6_F_5_I**), and 158.17° (**3‐*t*Bu⋅C_6_F_5_I**); F1‐I1‐C31: 179.63° (**1‐*t*Bu⋅C_6_F_5_I**), 179.34° (**2 b**–***t*** **Bu⋅C_6_F_5_I**), and 178.60° (**3‐*t*Bu⋅C_6_F_5_I**). Furthermore, the C_6_F_5_I molecule is more bent with respect to the pincer complex in **2‐*t*Bu⋅C_6_F_5_I** and **3‐*t*Bu⋅C_6_F_5_I** compared with that in **1‐*t*Bu⋅C_6_F_5_I**.

The computed structure of **1‐*t*Bu⋅C_6_F_5_I** can also be used to understand the observed trend in HOESY experiments (Figure 5). The shortest average distances between the *t*Bu protons (on P) and F (on C_6_F_5_I), *o*‐F, *m*‐F, *p*‐F, are 5.257 (Table S16 o, Supporting Information), 7.769 (Table S16 m), and 9.536 Å (Table S16 p) respectively, which is in line with the different intensities of cross peaks, *o*‐F⋅⋅⋅H^*t*Bu^>*m*‐F⋅⋅⋅H^*t*Bu^>*p*‐F⋅⋅⋅H^*t*Bu^, in the HOESY (Figure 5). For the co‐crystallised adduct **2‐*t*Bu⋅I‐C_6_F_4_‐I**, the shortest calculated average distances between the *t*Bu protons (on P) and F (on C_6_F_5_I), are 4.557 (for F *ortho* to I(1) and 5.443 Å (for F *ortho* to I(2)) (Table S17 a and S17 b).^27^ Computed structural parameters for these adducts are in the same range as found before for related halogen bonding systems with Group 10 fluoride complexes.11b

## Conclusion

We have for the first time synthesised a full series of isostructural square‐planar Group 10 metal(II) pincer monofluoride complexes. Along with this, a set of palladium fluoride complexes that differ only by substitution of the aryl backbone of the POCOP pincer ligand were prepared. In toluene solution, these complexes form strong halogen bonds with iodopentafluorobenzene in 1:1 stoichiometry. Thermodynamic parameters determined from ^19^F NMR titration experiments show only minor variations in Δ*H* ^o^ for the isostructural series of complexes **1‐*t*Bu**, **2‐*t*Bu** and **3‐*t*Bu**. However, binding constants *K* were much higher for Pd than for Ni and Pt complexes, suggesting that Pd complexes behave differently in halogen‐bonding systems. Enthalpic and entropic contributions to the halogen‐bonding interaction are consistently smaller for pincer systems described in this study compared to those previously reported for metal fluorides with perfluorinated pyridyl ligands (Figure 1, −23<Δ*H* ^0^<−16 kJ mol^−1^; −73<Δ*S* ^0^<−39 J K^−1^ mol^−1^).11b, 12 The structure of halogen‐bonding adducts was exemplarily analysed by co‐crystallisation of a Pd fluoride complex with 1,4‐diiodotetrafluorobenzene. In line with previous results on metal fluoride complexes8e this adduct shows an almost linear arrangement of XB donor and acceptor that is different from common strongly bent halogen bonds of heavier halides. The results presented herein could stimulate others in the field to systematically investigate related organometallic and metal‐free systems to gain further insights into the principles of halogen‐bonding interactions.

## Conflict of interest

The authors declare no conflict of interest.

## Supporting information

As a service to our authors and readers, this journal provides supporting information supplied by the authors. Such materials are peer reviewed and may be re‐organized for online delivery, but are not copy‐edited or typeset. Technical support issues arising from supporting information (other than missing files) should be addressed to the authors.

Supplementary
