# Supplementary material for: A Comparative Study on the Thermodynamics of Halogen Bonding of Group 10 Pincer Fluoride Complexes
Source: Chemistry. 2020 Feb 28;26(16):3571–7. doi: 10.1002/chem.201904863 (PMC7154528; doi:10.1002/chem.201904863)
Supplement: Supplementary file 1 — Supplementary [file CHEM-26-3571-s001.pdf]

CHEMISTRY
A European Journal

Supporting Information

# A Comparative Study on the Thermodynamics of Halogen Bonding of Group 10 Pincer Fluoride Complexes

Markus Joksch,[a] Hemlata Agarwala,[a, b] Monica Ferro,[a, c] Dirk Michalik,[a, d] Anke Spannenberg,[a] and Torsten Beweries*[a]

chem_201904863_sm_miscellaneous_information.pdf

Supporting Information

# A comparative study on the thermodynamics of halogen bonding of group 10 pincer fluoride complexes

Markus Joksch[1], Hemlata Agarwala[1,2], Monica Ferro[1,3], Dirk Michalik[1,4], Anke Spannenberg[1], Torsten Beweries[1]*

[1] *Leibniz-Institut für Katalyse e.V. an der Universität Rostock, Albert-Einstein-Straße 29a, 18059 Rostock, Germany, Email: torsten.beweries@catalysis.de*

[2] *Department of Synthetic Molecular Chemistry, Ångström Laboratory, Box 523, Uppsala University, Lägerhyddsvägen 1, 751 20 Uppsala, Sweden*

[3] *Politecnico di Milano, Dipartimento di Chimica, Materiali e Ing. Chimica "G. Natta", Via L. Mancinelli 7, 20131 Milano, Italy*

[4] *Universität Rostock, Institut für Chemie, Albert-Einstein-Str. 3a, 18059 Rostock, Germany*

**Table of contents**

S2 Experimental details

S5 NMR spectra of metal fluoride complexes

S8 Crystallographic details

S11 NMR titration experiments and analysis of the data

S17 Job plots

S18 Temperature calibration of the NMR spectrometer

S19 Computational details

## Experimental Details

### *General information*

All manipulations were carried out in an oxygen- and moisture-free argon atmosphere using standard Schlenk and glovebox techniques. Toluene and *n*-hexane were dispensed from a solvent purification system (PureSolv, Innovative Technology) and stored under Argon. Toluene-$d_8$ was dried over sodium, freshly distilled prior to use and stored under Argon. Iodopentafluorobenzene and 1,4-diiodotetrafluorobenzene were purchased from Sigma-Aldrich and used as received. The synthesis of complex **2-H** was described before.[1] Group 10 metal chloride and iodide complexes used for the synthesis of **1-*t*Bu**, **2-*t*Bu**, **2-COOMe**, and **3-*t*Bu** were prepared according to published procedures.[2] NMR spectra were recorded on Bruker AV 300 or AV 400 spectrometers. All $^1$H and $^{13}$C NMR spectra are referenced using the chemical shifts of residual protio solvent resonances (toluene-$d_8$: $\delta^1$H 2.08, 6.97, 7.01, 7.09, $\delta^{13}$C 20.4, 125.1, 127.9, 128.9, 137.5). Chemical shifts are reported in ppm ($\delta$) relative to tetramethylsilane. The $^{31}$P{$^1$H} NMR spectra were referenced to external $H_3PO_4$ and the $^{19}$F NMR spectra to $CFCl_3$. $^{13}$C NMR data were extracted from $^1$H,$^{13}$C HMBC spectra. The $^{19}$F,$^1$H HOESY spectrum of **1-*t*Bu·$C_6F_5I$** was recorded on a Bruker AV 500 spectrometer in toluene-$d_8$, delay 10s, mixing time 1.7 s. Mass spectra were recorded on a MAT 95XP Thermo Fisher Mass Spectrometer using Electrospray Ionization mode. Elemental analysis was performed on a Leco TruSpec Micro CHNS analyser.

### *Synthesis of complexes*

**Synthesis of complex 1-*t*Bu:** Complex [(3,5-*t*Bu$_2$-$^{tBu}$POCOP$^{tBu}$)NiCl] (285 mg, 0.47 mmol) and AgF (299 mg, 2.36 mmol) were dried overnight in vacuum at 60 °C (partial sublimation can occur). Afterwards 10 mL of toluene were added and the suspension was stirred at 60 °C for 22 h. After filtration a clear, yellow solution was obtained from which the solvent was removed in vacuum to precipitate the desired compound. Traces of the FHF adduct can be removed by treatment with NaH, yielding complex **1-*t*Bu** (160 mg, 0.27 mmol, 58%). Single crystals suitable for an X-ray analysis were obtained by slow cooling of a hot toluene solution. $^1$H NMR (toluene-$d_8$, 300 MHz, 297 K): $\delta$ 7.10 (t, *J* = 1.2 Hz, 1H, *p*-Ar-H), 1.48 (vt, *J* = 7.0 Hz, 36H, P(C(CH$_3$)$_3$)$_2$), 1.44 (s, 18H, *m*-Ar-C(CH$_3$)$_3$). $^{13}$C NMR (toluene-$d_8$, 75 MHz, 297 K): $\delta$ 165.9 (s, C$_{Ar}$-O-P), 126.5 (t, *J* = 4.9 Hz, *m*-C$_{Ar}$), 125.5 (Ni-C), 123.4 (s, *p*-C$_{Ar}$), 38.3 (t, *J* = 6.6 Hz, P(*C*(CH$_3$)$_3$)$_2$), 34.4 (s, *m*-Ar-*C*(CH$_3$)$_3$), 30.5 (s, *m*-Ar-C(*C*H$_3$)$_3$), 27.9 (t, *J* = 3.1 Hz, P(C(*C*H$_3$)$_3$)$_2$). $^{31}$P NMR (toluene-$d_8$, 121 MHz, 297 K): $\delta$ 179.1 (d, *J* = 27.0 Hz). $^{19}$F NMR (toluene-$d_8$, 282 MHz, 297 K): $\delta$ -380.1 (d, *J* = 27.0 Hz). CH analysis: Anal. calcd. for $C_{30}H_{55}FO_2P_2Ni$: C, 61.34; H,

9.44. Found: C, 60.20; H, 9.17. Due to the highly hygroscopic nature of **1-*t*Bu** no accurate values could be obtained. MS (CI positive, isobutane): *m/z* 586 $[M]^+$, 567 $[M-F]^+$.

**Synthesis of complex 2-*t*Bu:** Complex [(3,5-*t*Bu-$^{tBu}$POCOP$^{tBu}$)PdI] (312 mg, 0.42 mmol) and AgF (266 mg, 2.10 mmol) were dried overnight in vacuum at 80 °C (partial sublimation can occur). Afterwards 10 mL of toluene were added and the suspension was stirred at room temperature for 18 h. After filtration a clear, pale red solution was obtained. The solvent was removed and the material was washed with 5 mL of cyclohexane. Further purification was done by crystallisation from toluene yielding the pale yellow complex **2-*t*Bu** (162 mg, 0.26 mmol, 61%). Colourless single crystals suitable for an X-ray analysis were obtained by slow cooling of a hot toluene solution. $^1$H NMR (toluene-$d_8$, 300 MHz, 297 K): $\delta$ 7.10 (t, *J* = 1.2 Hz, 1H, *p*-Ar-H), 1.48 (vt, *J* = 7.0 Hz, 36H, $P(C(CH_3)_3)_2$), 1.44 (s, 18H, *m*-Ar-$C(CH_3)_3$). $^{13}$C NMR (toluene-$d_8$, 75 MHz, 297 K): $\delta$ 163.7 (s, $C_{Ar}$-O-P), 132.3 (Pd-C), 127.4 (br s, *m*-$C_{Ar}$), 122.6 (s, *p*-$C_{Ar}$), 39.0 (t, *J* = 7.5 Hz, $P(\mathit{C}(CH_3)_3)_2$), 34.7 (s, *m*-Ar-$\mathit{C}(CH_3)_3$), 30.5 (s, *m*-Ar-$C(CH_3)_3$), 27.7 (t, *J* = 4.2 Hz, $P(C(\mathit{C}H_3)_3)_2$). $^{31}$P NMR (toluene-$d_8$, 121 MHz, 297 K): $\delta$ 185.6 (s). $^{19}$F NMR (toluene-$d_8$, 282 MHz, 297 K): $\delta$ -315.6 (s). CH analysis: Anal. calcd. for $C_{30}H_{55}FO_2P_2Pd$: C, 56.73; H, 8.73. Found: C, 56.76; H, 8.58. MS (CI positive, isobutane): *m/z* 634 $[M]^+$, 615 $[M-F]^+$.

Palladium fluoride complexes are highly sensitive towards moisture. In the presence of traces of water complex **2-*t*Bu** crystallises as the water adduct **2-*t*Bu·H₂O**. The molecular structure (Figure S7) of this adduct was analysed using material that was recrystallised from toluene.

**Synthesis of complex 2-COOMe:** Complex [(3,5-COOMe$_2$-$^{tBu}$POCOP$^{tBu}$)PdI] (186 mg, 0.25 mmol) and AgF (158 mg, 1.25 mmol) were dried overnight in vacuum at 50 °C (partial sublimation can occur). Afterwards 11 mL of toluene were added and the suspension was stirred at room temperature for 18 h. After filtration a clear and colourless solution was obtained, from which the solvent was removed in vacuum to precipitate the desired compound **2-COOMe** (110 mg, 0.17 mmol, 69%). Single crystals suitable for an X-ray analysis were obtained by slow cooling of a hot toluene solution. $^1$H NMR (toluene-$d_8$, 300 MHz, 297 K): $\delta$ 8.64 (t, *J* = 1.1 Hz, 1H, *p*-Ar-H), 3.49 (s, 6H, $COOCH_3$), 1.34 (vt, *J* = 7.6 Hz, 36H, $P(C(CH_3)_3)_2$). $^{13}$C NMR (toluene-$d_8$, 75 MHz, 297 K): $\delta$ 169.3 (t, *J* = 6.6 Hz, $C_{Ar}$-O-P), 164.4 (s, $\mathit{C}OOCH_3$), 134.5 (s, *p*-$C_{Ar}$), 132.9 (s, Pd-C), 111.2 (t, *J* = 6.1 Hz, *m*-$C_{Ar}$), 51.2 (s, $COO\mathit{C}H_3$), 39.1 (t, *J* = 7.2 Hz, $P(\mathit{C}(CH_3)_3)_2$), 27.2 (t, *J* = 4.2 Hz, $P(C(\mathit{C}H_3)_3)_2$). $^{31}$P NMR (toluene-$d_8$, 121 MHz, 297 K): $\delta$ 191.0 (s). $^{19}$F NMR (toluene-$d_8$, 282 MHz, 297 K): $\delta$ -326.3 (s). CH analysis: Anal. calcd. for $C_{26}H_{43}FO_6P_2Pd$: C, 48.87; H, 6.78. Found: C, 49.62; H, 6.82. Despite extensive drying of samples for CH analysis in vacuum no accurate values could be obtained for **2-COOMe**. MS (CI positive, isobutane): *m/z* 619 $[M-F]^+$.

**Synthesis of complex 3-*t*Bu:** Complex [(3,5-*t*Bu-$^{tBu}$POCOP$^{tBu}$)PtI] (470 mg, 0.57 mmol) and AgF (717 mg, 5.65 mmol) were dried overnight in vacuum at 60 °C (partial sublimation can occur). Afterwards 18 mL of toluene were added and the suspension was stirred at room temperature for 18 h. The mixture was filtered and treated with AgF (72 mg, 0.57 mmol) again. After filtration a clear and colourless solution was obtained. The solvent was reduced to 2 mL and 3 mL of *n*-hexane were added. The mixture was stored at room temperature and after several hours a colourless precipitate formed that could be isolated by filtration. Traces of the FHF adduct can be removed by treatment with NaH, yielding complex **3-*t*Bu** (262 mg, 0.36 mmol, 64%). $^{1}$H NMR (toluene-$d_8$, 300 MHz, 297 K): $\delta$ 7.14 (t, *J* = 1.2 Hz, 1H, *p*-Ar-H), 1.47 (s, 18H, *m*-Ar-C(CH$_3$)$_3$), 1.40 (vt, *J* = 7.5 Hz, 36H, P(C(CH$_3$)$_3$)$_2$). $^{13}$C NMR (toluene-$d_8$, 75 MHz, 297 K): $\delta$ 162.6 (s, C$_{Ar}$-O-P), 127.0 (t, *J* = 4.8 Hz, *m*-C$_{Ar}$), 121.2 (s, *p*-C$_{Ar}$), 117.5 (Pt-C), 40.2 (t, *J* = 12.1 Hz, P(*C*(CH$_3$)$_3$)$_2$), 34.6 (s, *p*-Ar-*C*(CH$_3$)$_3$), 30.5 (s, *p*-Ar-C(*C*H$_3$)$_3$), 27.7 (t, *J* = 3.6 Hz, P(C(*C*H$_3$)$_3$)$_2$). $^{31}$P NMR (toluene-$d_8$, 121 MHz, 297 K): $\delta$ 174.3 (d, $J_{P,F}$ = 2.0 Hz); 174.3 (dd, $J_{Pt,P}$ = 3215 Hz, $J_{P,F}$ = 2.0 Hz). $^{19}$F NMR (toluene-$d_8$, 282 MHz, 297 K): $\delta$ -309.9 (s); -309.9 (d, $J_{Pt,F}$ = 136.0 Hz). CH analysis: Anal. calcd. for $C_{30}H_{55}FO_2P_2Pt$: C, 49.79; H, 7.66. Found: C, 49.73; H, 7.60. MS (CI positive, isobutane): *m/z* 723 [M]$^+$, 704 [M-F]$^+$.

**Synthesis of adduct 2-*t*Bu·I-C$_6$F$_4$-I:** Complex **2-*t*Bu** (31.0 mg, 48.8 µmol) and $C_6F_4I_2$ (10.1 mg, 25.0 µmol) were suspended in 1.8 mL of toluene and heated to 100 °C. Upon slow cooling overnight crystals formed that were isolated by decanting.

## NMR spectra of metal fluoride complexes

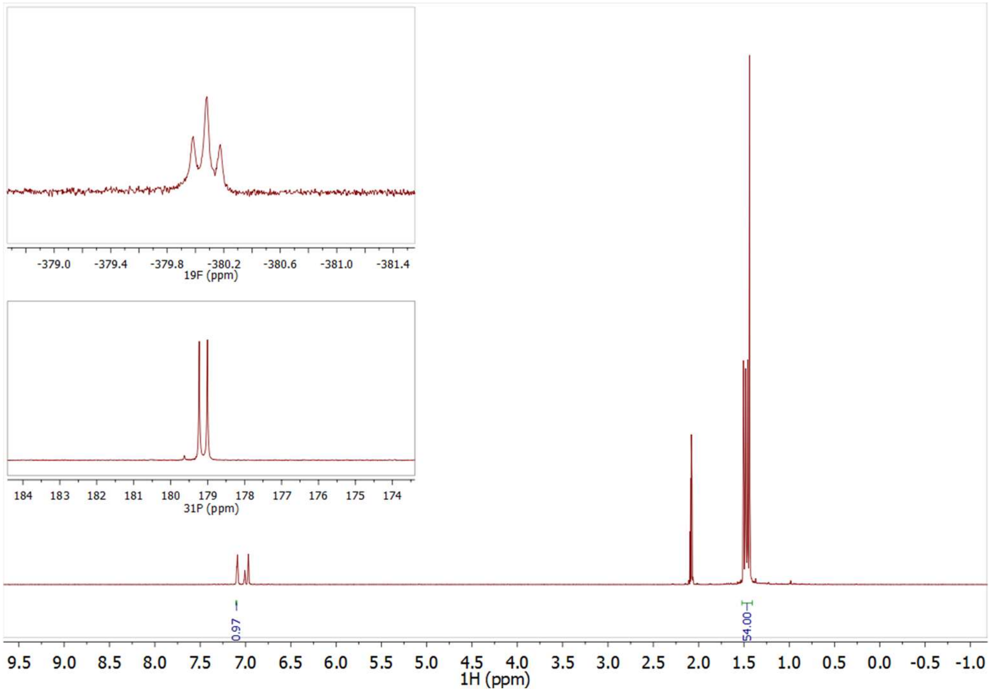


**Figure S1.** $^{1}H$, $^{19}F$ and $^{31}P$ NMR spectra of complex **1-*t*Bu** (toluene-$d_8$, 297 K).

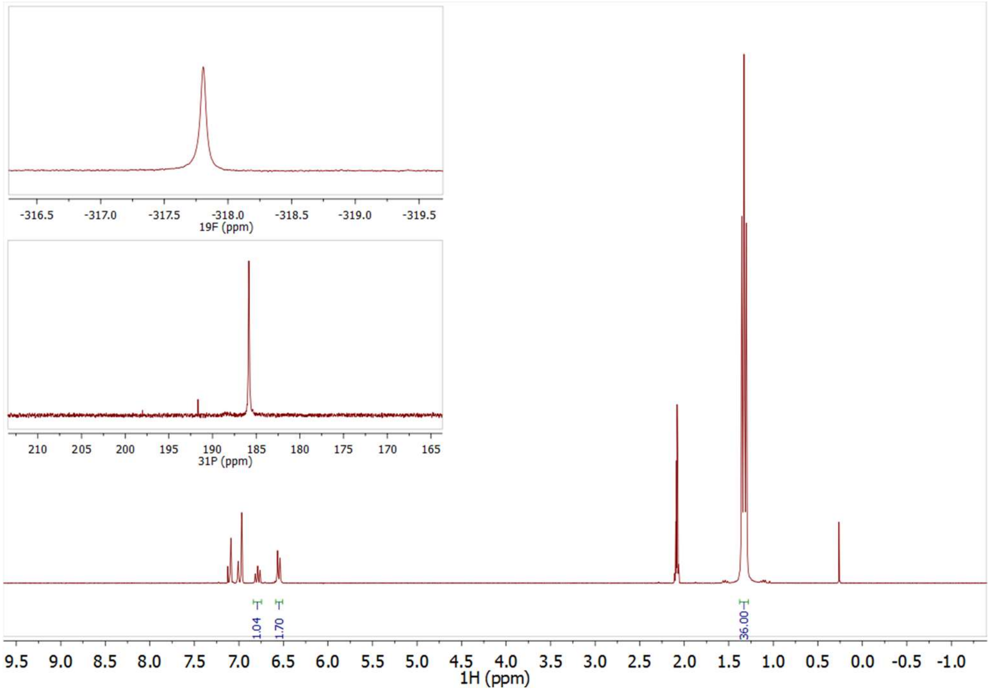


**Figure S2.** $^{1}H$, $^{19}F$ and $^{31}P$ NMR spectra of complex **2-H** (toluene-$d_8$, 297 K). The $^{31}P$ resonance at 192.1 ppm corresponds to traces of the palladium chloride precursor that could not be removed by crystallisation.

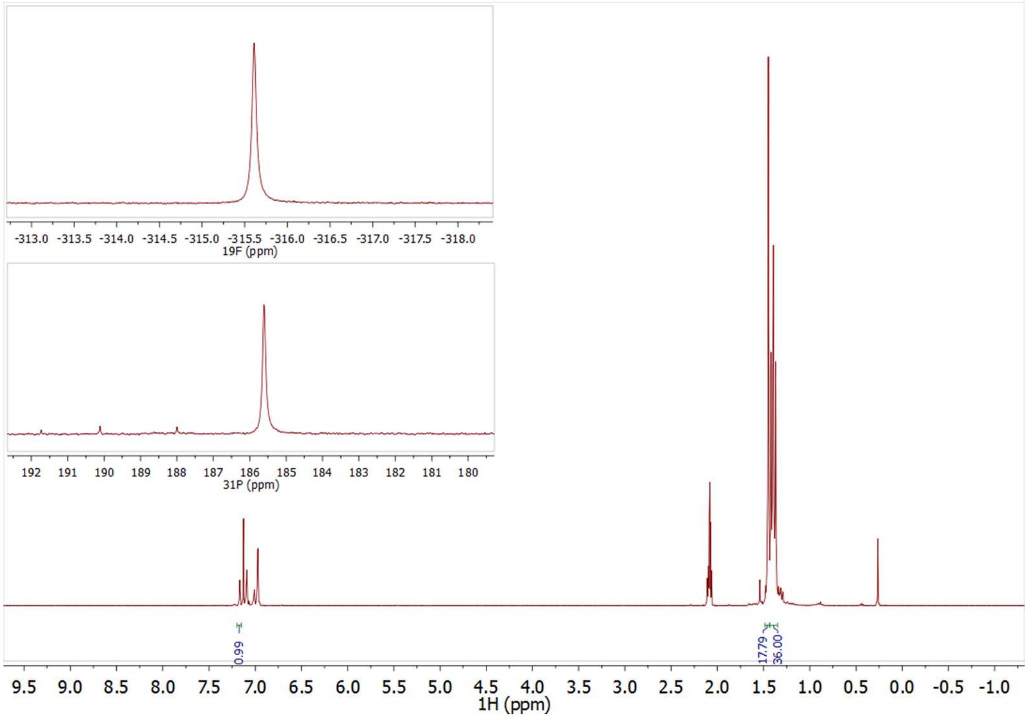


**Figure S3.** $^{1}H$, $^{19}F$ and $^{31}P$ NMR spectra of complex **2-*t*Bu** (toluene-$d_8$, 297 K).

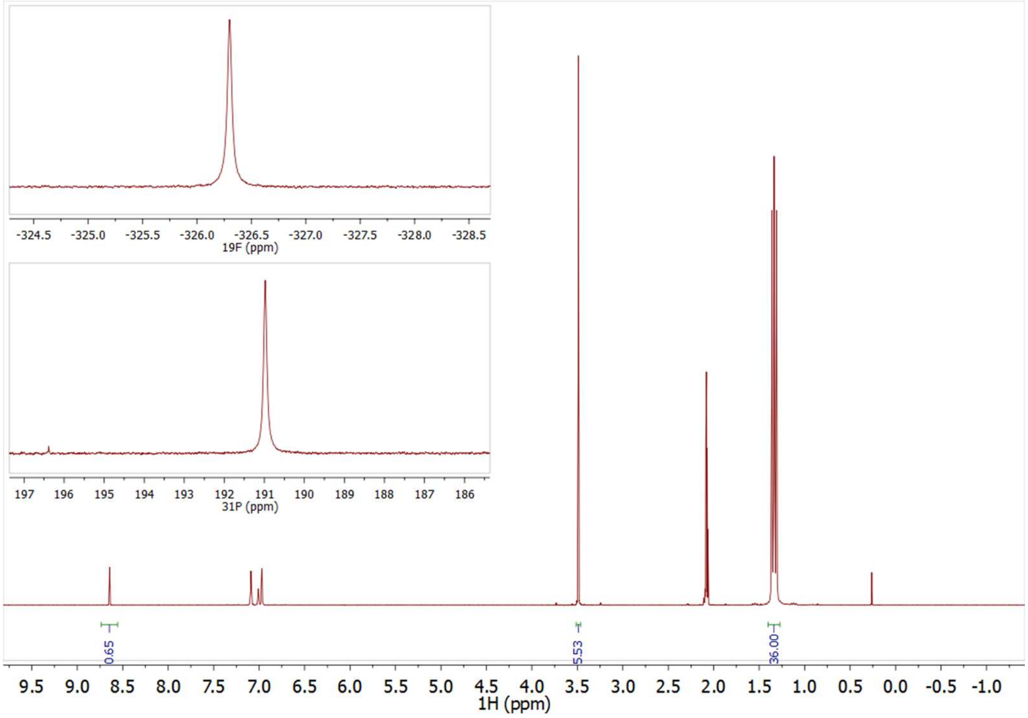


**Figure S4.** $^{1}H$, $^{19}F$ and $^{31}P$ NMR spectra of complex **2-COOMe** (toluene-$d_8$, 297 K).

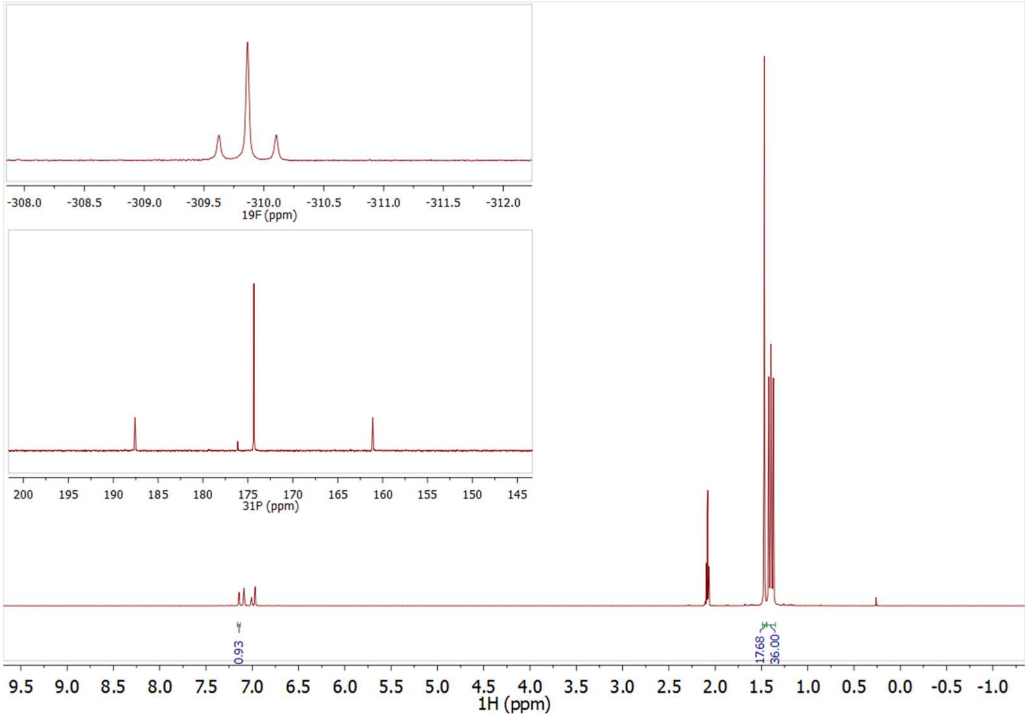


**Figure S5.** $^{1}$H, $^{19}$F and $^{31}$P NMR spectra of complex **3-*t*Bu** (toluene-*d*$_8$, 297 K). The $^{31}$P resonance at 176.1 ppm corresponds to traces of the platinum chloride precursor that could not be removed by crystallisation.

## Crystallographic details

Single crystal X-ray diffraction (SC-XRD) data were collected on a Bruker Kappa APEX II Duo diffractometer. The structures were solved by direct methods (SHELXS-97[3]) and refined by full-matrix least-squares procedures on $F^2$ (SHELXL-2014[4]). Diamond[5] and Mercury[6] were used for graphical representations. The data collected from crystals of **2-*t*Bu·I-$C_6F_4$-I** were refined as two-component twin. CCDC 1585243-1585245 and 1947526-1947528 contain the supplementary crystallographic data for this paper. These data are provided free of charge by The Cambridge Crystallographic Data Centre.

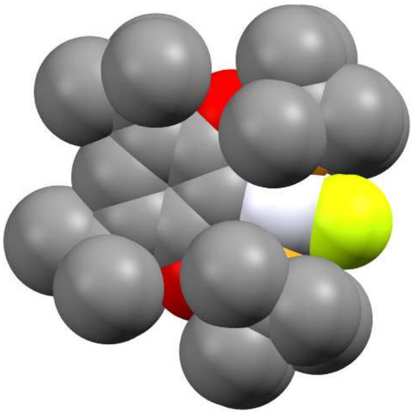

**Figure S6.** Spacefill model of complex **3-*t*Bu**, exemplarily showing the shielding of the M-F moiety by the *t*-Bu groups.

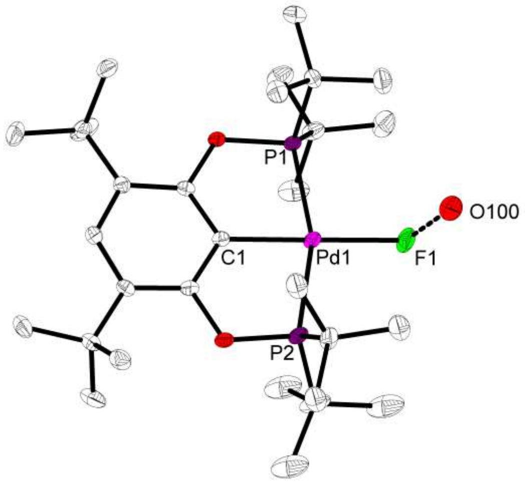


**Figure S7.** Molecular structure of complex **2-*t*Bu·$H_2O$**. Thermal ellipsoids correspond to 30% probability. Hydrogen atoms were omitted for clarity. The occupancy of co-crystallised $H_2O$ was fixed at 0.15 which is in line with NMR data that show traces of $H_2O$ (localisation of the O-bound H atoms was not possible). The minor components of the disordered POCOP ligand were removed for clarity.

**Table S1.** Crystallographic data.

| | **1-*t*Bu** | **2-*t*Bu** | **2-COOMe** |
|---|---|---|---|
| Chem. Formula | $C_{30}H_{55}FNiO_2P_2$ | $C_{30}H_{55}FO_2P_2Pd$ | $C_{26}H_{43}FO_6P_2Pd$ |
| Form. Wght [g mol$^{-1}$] | 587.39 | 635.08 | 638.94 |
| Colour | yellow | colourless | colourless |
| Cryst. system | monoclinic | monoclinic | monoclinic |
| Space group | $P2_1/c$ | $P2_1/c$ | $P2_1/n$ |
| a [Å] | 15.1675(6) | 11.2296(5) | 11.7507(11) |
| b [Å] | 18.3366(7) | 22.1635(11) | 20.7594(19) |
| c [Å] | 23.6291(10) | 14.0558(7) | 12.3434(12) |
| $\alpha$ [°] | 90 | 90 | 90 |
| β [°] | 98.422(2) | 109.0031(14) | 97.543(3) |
| γ [°] | 90 | 90 | 90 |
| V [Å$^3$] | 6500.9(5) | 3307.7(3) | 2985.0(5) |
| Z | 8 | 4 | 4 |
| $\rho_{calc.}$ [g cm$^{-3}$] | 1.200 | 1.275 | 1.422 |
| μ [mm$^{-1}$] | 2.013 | 0.686 | 0.771 |
| T [K] | 150(2) | 220(2) | 150(2) |
| radiation type | CuK$\alpha$ | MoK$\alpha$ | MoK$\alpha$ |
| reflections measured | 46941 | 71540 | 63695 |
| independent reflections | 10754 | 7983 | 7195 |
| observed reflections with I > 2σ(I) | 9780 | 6771 | 6496 |
| $R_{int.}$ | 0.0342 | 0.0345 | 0.0312 |
| F(000) | 2544 | 1344 | 1328 |
| $R_1$ ($I > 2\sigma(I)$]) | 0.0379 | 0.0339 | 0.0273 |
| $wR_2$ (all data) | 0.1013 | 0.0912 | 0.0659 |
| GOF on $F^2$ | 1.059 | 1.054 | 1.085 |
| Parameters | 685 | 419 | 339 |
| CCDC number | 1585243 | 1585244 | 1947527 |

| | **3-*t*Bu** | **2-*t*Bu·$H_2O$** | **2-*t*Bu·I-$C_6F_4$-I** |
|---|---|---|---|
| Chem. Formula | $C_{30}H_{55}FO_2P_2Pt$ | $C_{30}H_{55}FO_{2.15}P_2Pd$ | $C_{66}H_{110}F_6I_2O_4P_4Pd_2$ |
| Form. Wght [g mol$^{-1}$] | 723.77 | 637.48 | 1672.01 |
| Colour | colourless | colourless | colourless |
| Cryst. system | monoclinic | monoclinic | triclinic |
| Space group | $P2_1/c$ | $P2_1/c$ | $P\bar{1}$ |
| a [Å] | 11.3096(9) | 11.2046(5) | 12.2889(5) |
| b [Å] | 23.1457(17) | 22.0056(11) | 12.7819(6) |
| c [Å] | 13.2709(10) | 14.0312(7) | 14.1223(6) |
| $\alpha$ [°] | 90 | 90 | 110.9828(16) |
| β [°] | 110.1510(11) | 108.9562(15) | 113.7181(14) |
| γ [°] | 90 | 90 | 90.5675(15) |
| V [Å$^3$] | 3261.3(4) | 3272.0(3) | 1865.23(14) |
| Z | 4 | 4 | 1 |
| $\rho_{calc.}$ [g cm$^{-3}$] | 1.474 | 1.294 | 1.489 |
| μ [mm$^{-1}$] | 4.430 | 0.694 | 1.452 |
| T [K] | 150(2) | 150(2) | 150(2) |
| radiation type | MoK$\alpha$ | MoK$\alpha$ | MoK$\alpha$ |
| reflections measured | 42955 | 36443 | 74804 |
| independent reflections | 7876 | 7129 | 8137 |
| observed reflections with $I > 2\sigma(I)$ | 6918 | 6477 | 7081 |
| $R_{int.}$ | 0.0280 | 0.0212 | 0.0474 |
| F(000) | 1472 | 1349 | 850 |
| $R_1$ ($I > 2\sigma(I)$]) | 0.0210 | 0.0309 | 0.0283 |
| $wR_2$ (all data) | 0.0440 | 0.0771 | 0.0652 |
| GOF on $F^2$ | 1.094 | 1.042 | 1.046 |
| Parameters | 343 | 347 | 398 |
| CCDC number | 1947528 | 1947526 | 1585245 |

## NMR titration experiments and analysis of the data

### *General information*

Equilibrium constants were determined by $^{19}$F NMR titration at different temperatures by monitoring the chemical shift of the metal fluoride. For simplicity, the volumes of the solutions were assumed to be the sum of the volumes of the components, thus allowing for calculation of the densities of the solutions. The activities of the components were assumed equal to their molar concentration. Calculations of the equilibrium constants were done using a Microsoft Excel macro programmed by Prof. Christopher A. Hunter (University of Cambridge). Two parameters were fitted, namely the equilibrium constant *K* and the downfield shift from the signal of free metal fluoride for the coordinated fluoride in the adduct, $\Delta\delta_{max}$. Both parameters can be conveniently fitted using a 1:1 binding model for the whole range of temperatures without using restraints. $\Delta H$ and $\Delta S$ were calculated from the Van't Hoff plots of the equilibrium constants. Samples for each titration experiment were prepared freshly as follows: Two individual solutions of known concentration of the metal fluoride and the donor were prepared by dissolving the complex and $C_6F_5I$ in toluene (Table S2). Approximately equal amounts of the solution of the complex were transferred into each of ten labelled NMR tubes in the glove box. The exact mass of the solution contained in each tube was recorded, followed by addition of varying amounts (approximately from 0-20 equivalents) to the tubes and registration of the exact mass. $^{19}$F NMR spectra of all samples were recorded at various temperatures. The samples were kept in a bath close to the temperature of the NMR probe and left to equilibrate inside the NMR spectrometer before the spectrum was recorded. Spectra were recorded multiple times to exclude reactions of XB donor and acceptor. The $^{19}$F NMR spectra were collected unlocked as for each temperature the spectrometer was shimmed with a solution of the metal fluoride in toluene-$d_8$ and maintained with the same settings throughout this temperature.

### *Composition of stock solutions*

**Table S2.** Composition of stock solution used for the preparation of NMR samples.

| *Titration experiment* | **metal fluoride solution** | | **$C_6F_5I$ solution** | |
|---|---|---|---|---|
| | **m(metal fluoride) [mg]** | **m(toluene) [mg]** | **m($C_6F_5I$) [mg]** | **m(toluene) [mg]** |
| **1-*t*Bu**/$C_6F_5I$ | 32.8 | 5506.0 | 185.1 | 904.8 |
| **2-H**/$C_6F_5I$ | 33.8 | 5502.0 | 181.3 | 900.4 |
| **2-*t*Bu**/$C_6F_5I$ | 36.5 | 5497.2 | 185.7 | 901.9 |
| **2-COOMe**/$C_6F_5I$ | 37.6 | 5581.7 | 184.4 | 907.4 |
| **3-*t*Bu**/$C_6F_5I$ | 40.0 | 5507.1 | 187.3 | 901.0 |

***Titration curves and equilibrium constants***

*Complex **1-tBu**/$C_6F_5I$*

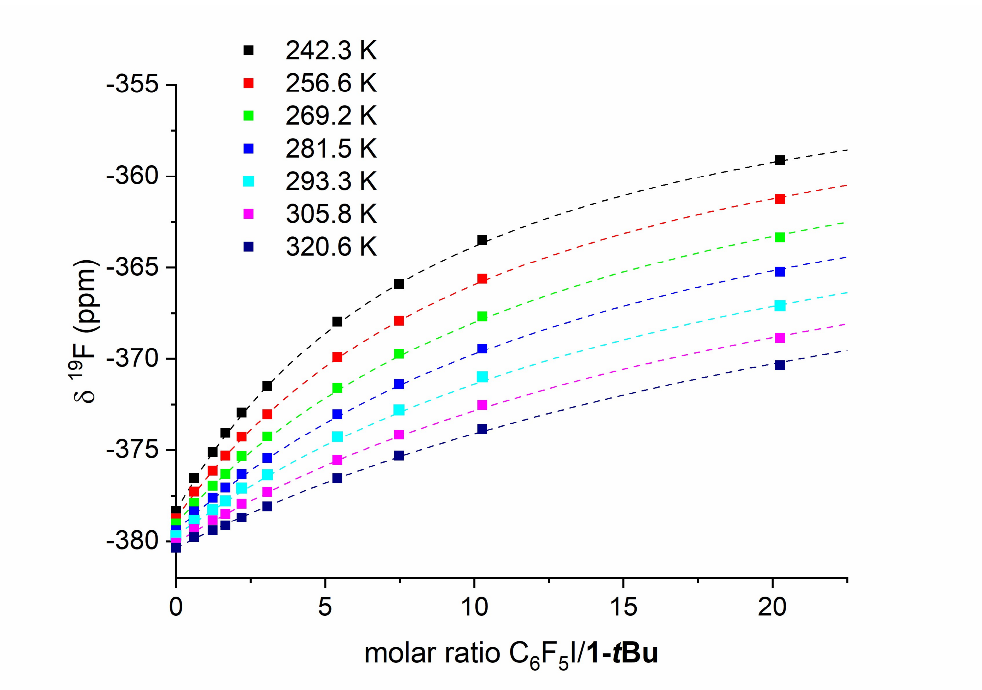


**Figure S8.** Titration data at different temperatures, showing values for the $^{19}F$ chemical shift of the metal fluoride δF vs. molar ratio of $C_6F_5I$ and complex **1-*t*Bu**. Squares are experimental data, dotted lines correspond to the best fit to a 1:1 binding isotherm.

**Table S3.** Binding constants for 1:1 binding of complex **1-*t*Bu** and $C_6F_5I$. $\Delta\delta$ corresponds to the difference between the experimental chemical shift of the free metal fluoride and the fitted chemical shift of the 1:1 adduct. Values for $\Delta\delta$ were fixed to 33 ppm during fitting for the two highest temperatures.

| ***T* in K** | **$\Delta\delta$ in ppm** | ***K*** |
|---|---|---|
| 242.3 | 31.8 | 11.8(1) |
| 256.6 | 32.3 | 9.1(1) |
| 269.2 | 32.8 | 7.0(1) |
| 281.5 | 33.2 | 5.66(8) |
| 293.3 | 33.3 | 4.64(6) |
| 305.8 | 33.0 | 3.88(4) |
| 320.6 | 33.0 | 3.20(4) |

*Complex **2-H**/$C_6F_5I$*

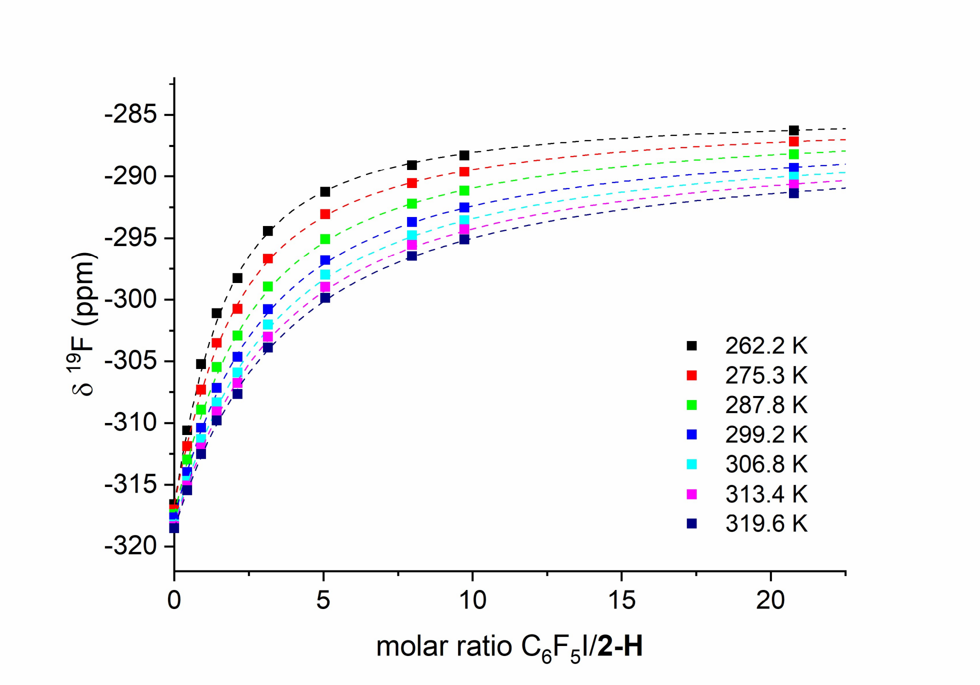


**Figure S9.** Titration data at different temperatures, showing values for the $^{19}F$ chemical shift of the metal fluoride δF vs. molar ratio of $C_6F_5I$ and complex **2-H**. Squares are experimental data, dotted lines correspond to the best fit to a 1:1 binding isotherm. Due to limited solubility of complex **2-H** no titration data for 242 K are given.

**Table S4.** Binding constants for 1:1 binding of complex **2-H** and $C_6F_5I$. $\Delta\delta$ corresponds to the difference between the experimental chemical shift of the free metal fluoride and the fitted chemical shift of the 1:1 adduct.

| ***T* in K** | **$\Delta\delta$ in ppm** | ***K*** |
|---|---|---|
| 262.2 | 32.3 | 93(1) |
| 275.3 | 32.8 | 67(1) |
| 287.8 | 32.9 | 51.3(8) |
| 299.2 | 33.1 | 40.5(6) |
| 306.8 | 33.5 | 36.5(4) |
| 313.4 | 33.6 | 32.7(3) |
| 319.6 | 33.5 | 29.7(3) |

*Complex **2-tBu**/$C_6F_5I$*

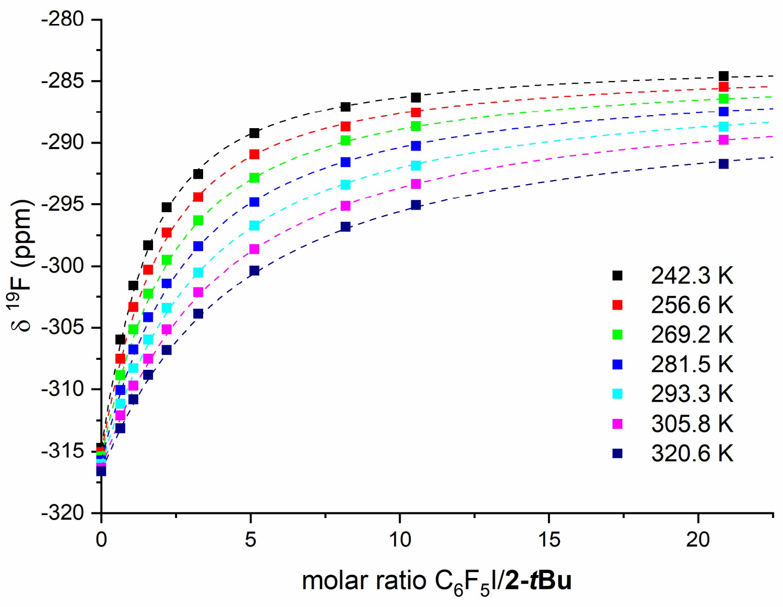


**Figure S10.** Titration data at different temperatures, showing values for the $^{19}F$ chemical shift of the metal fluoride δF vs. molar ratio of $C_6F_5I$ and complex **2-*t*Bu**. Squares are experimental data, dotted lines correspond to the best fit to a 1:1 binding isotherm.

**Table S5.** Binding constants for 1:1 binding of complex **2-*t*Bu** and $C_6F_5I$. $\Delta\delta$ corresponds to the difference between the experimental chemical shift of the free metal fluoride and the fitted chemical shift of the 1:1 adduct.

| *T* in K | $\Delta\delta$ in ppm | *K* |
|---|---|---|
| 242.3 | 31.7 | 116(1) |
| 256.6 | 31.6 | 90(1) |
| 269.2 | 31.9 | 68.4(7) |
| 281.5 | 31.9 | 53.6(6) |
| 293.3 | 31.9 | 41.8(5) |
| 305.8 | 32.2 | 33.1(4) |
| 320.6 | 31.3 | 29.0(3) |

*Complex **2-COOMe**/$C_6F_5I$*

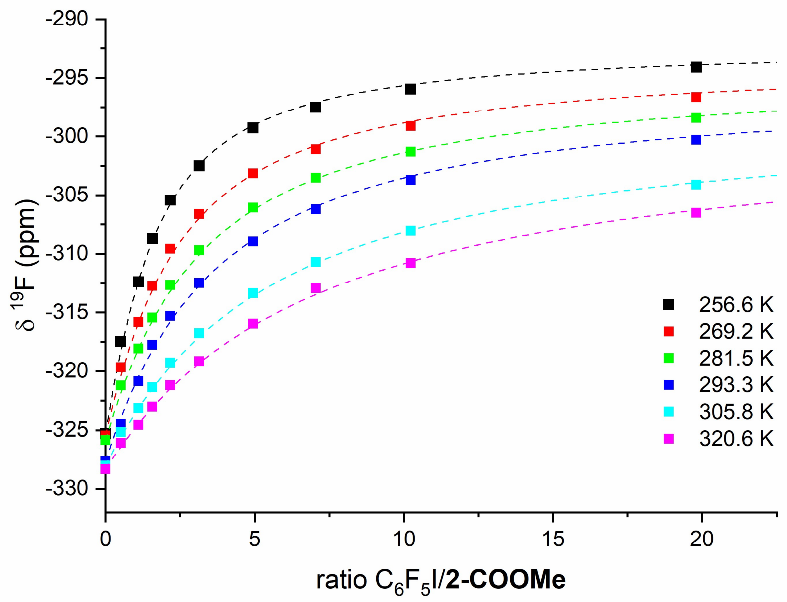


**Figure S11.** Titration data at different temperatures, showing values for the $^{19}F$ chemical shift of the metal fluoride δF vs. molar ratio of $C_6F_5I$ and complex **2-COOMe**. Squares are experimental data, dotted lines correspond to the best fit to a 1:1 binding isotherm. Due to limited solubility of complex **2-COOMe** no titration data for 242 K are given.

**Table S6.** Binding constants for 1:1 binding of complex **2-COOMe** and $C_6F_5I$. $\Delta\delta$ corresponds to the difference between the experimental chemical shift of the free metal fluoride and the fitted chemical shift of the 1:1 adduct.

| ***T* in K** | **$\Delta\delta$ in ppm** | ***K*** |
|---|---|---|
| 256.6 | 33.2 | 105(2) |
| 269.2 | 32.1 | 64(2) |
| 281.5 | 32.2 | 44.2(8) |
| 293.3 | 33.1 | 35.7(7) |
| 305.8 | 32.1 | 22.2(4) |
| 320.6 | 31.9 | 16.6(3) |

*Complex **3-tBu**/$C_6F_5I$*

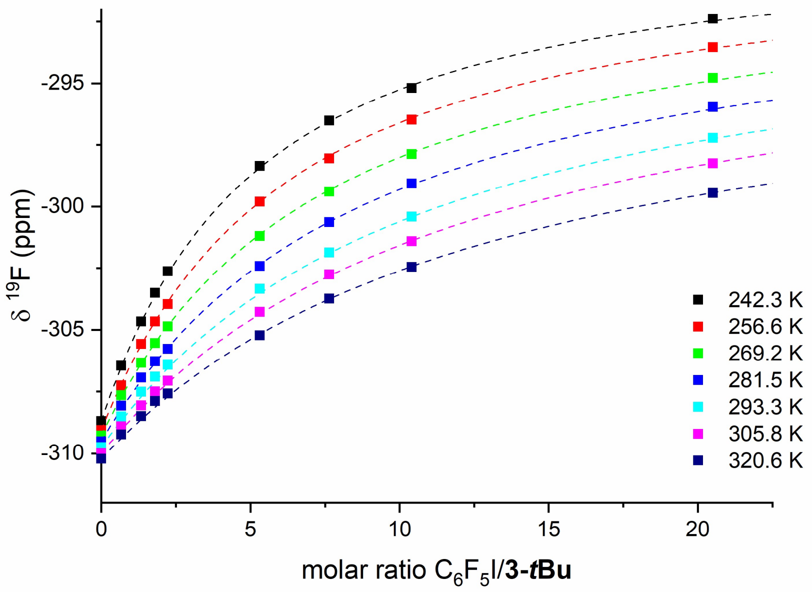


**Figure S12.** Titration data at different temperatures, showing values for the $^{19}$F chemical shift of the metal fluoride δF vs. molar ratio of $C_6F_5I$ and complex **3-*t*Bu**. Squares are experimental data, dotted lines correspond to the best fit to a 1:1 binding isotherm.

**Table S7.** Binding constants for 1:1 binding of complex **3-*t*Bu** and $C_6F_5I$. $\Delta\delta$ corresponds to the difference between the experimental chemical shift of the free metal fluoride and the fitted chemical shift of the 1:1 adduct.

| ***T* in K** | **$\Delta\delta$ in ppm** | ***K*** |
|---|---|---|
| 242.3 | 21.4 | 24.1(2) |
| 256.6 | 21.9 | 18.5(2) |
| 269.2 | 21.9 | 15.0(2) |
| 281.5 | 22.1 | 12.1(2) |
| 293.3 | 21.4 | 10.6(1) |
| 305.8 | 21.7 | 8.9(1) |
| 320.6 | 20.7 | 8.1(1) |

***Job plots***

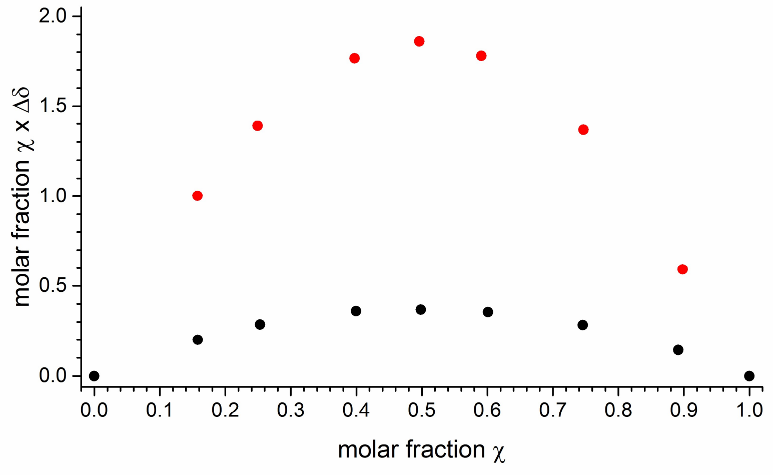


**Figure S13.** Qualitative comparison of Job plots derived from mixtures of $C_6F_5I$ and complexes **1-*t*Bu** (black) and **2-COOMe** (red), respectively. Stronger binding in case of the Pd complex is evidenced by the larger amplitude of the curve (see Figure SX for a comparison).

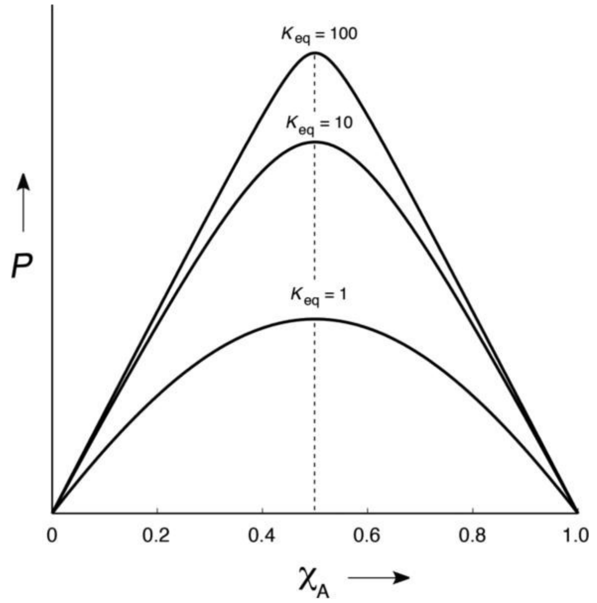


**Figure S14.** Literature example of a Job plot for three systems with moderate ($K_{eq}$ = 1), strong ($K_{eq}$ = 10) and very strong binding ($K_{eq}$ = 100).[7]

## Temperature calibration of the NMR spectrometer

The temperature of the NMR probe was corrected using a procedure described earlier (Table S8).[8] All other temperatures were determined from a linear regression plot of set vs. calculated data.

**Table S8.** Chemical shifts of MeOH used for temperature correction of the NMR data.

| **δ OH (ppm)** | **δ $CH_3$ (ppm)** | **Δδ (ppm)** | **set T (K)** | **calculated T (K)** |
|---|---|---|---|---|
| 5.36659 | 3.3299 | 2.04 | 253.0 | 242.3 |
| 5.2581 | 3.329 | 1.92 | 263.0 | 256.6 |
| 5.14 | 3.33 | 1.81 | 273.0 | 269.2 |
| 5.0359 | 3.3308 | 1.7 | 283.0 | 281.5 |
| 4.9187 | 3.3297 | 1.59 | 293.0 | 293.3 |
| 4.7971 | 3.3301 | 1.47 | 303.0 | 305.8 |
| 4.6438 | 3.3291 | 1.32 | 313.0 | 320.6 |

## Computational details

All structures were optimised *in vacuo* and in toluene using Gaussian 09[9] program. Optimisations were done using B3PW91[10], BHandHLYP[11], M06[12], and B3LYP[10] functionals. Selected bond distances and angles were compared between the crystal structures and the DFT optimised structures, and their root mean square error (RMSE) values calculated according to the following equation:

$$\text{RMSE} = \sqrt{\frac{\sum_{i=1}^{n}\left(\frac{\left|d^{i}_{theoretical}-d^{i}_{experimental}\right|}{d^{i}_{experimental}}*100\right)^2+\sum_{j=1}^{m}\left(\frac{\left|\theta^{j}_{theoretical}-\theta^{j}_{experimental}\right|}{\theta^{j}_{experimental}}*100\right)^2}{n+m}}$$

{**RMSE** = root mean square of the percentage errors between each of the DFT calculated and the corresponding experimental bond distances (*d*) and angles (θ). It should be noted that the RMSE is dimensionless here}.

Since the B3LYP functional showed the best resemblance to the crystal geometry of **2-*t*Bu·I-$C_6F_4$-I**, considering only the bond distances and angles associated with the halogen bonding (Table S9a), it was chosen for discussion on the structures of the adducts with $C_6F_5I$. The basis set LANL2DZ[13] was used for the metal (Ni, Pd, Pt), P and I. 6-31G*[14] basis set was used for C, H, O. 6-31+G* was used for F atoms. Single point energy calculations were performed on all the optimised structures. Frequency calculations were done on the optimised structures to ascertain that there were no imaginary frequencies, thus confirming that the structure reached an energy minimum. The geometries of the optimised structures in gas phase were used as input geometries for optimisations in toluene as the solvent using the conductor-like polarisable continuum model (CPCM) [15]. All the optimised structures were viewed with GaussView 5 and Chemcraft.[16]

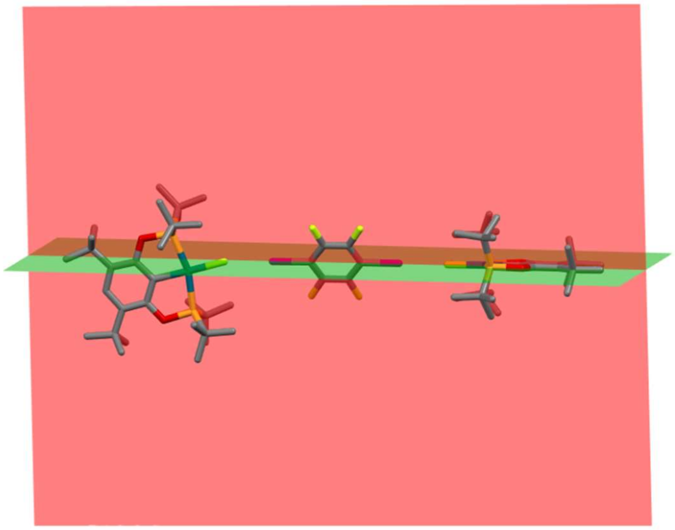

**Figure S15a.** DFT optimised structure of **2-*t*Bu·I-$C_6F_4$-I** in toluene, depicting the planes (coloured red and green) passing through the benzene rings of the two molecules of **2-*t*Bu**.

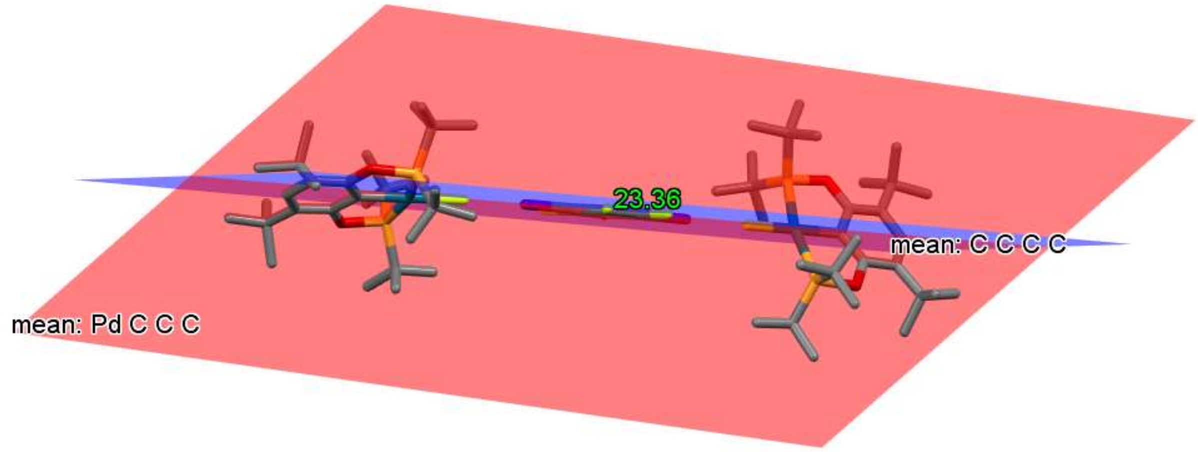


**Figure S15b.** DFT optimised structure of **2-*t*Bu·I-C$_6$F$_4$-I** in toluene, depicting the planes (coloured red and blue) passing through the benzene ring of one of the two molecules of **2-*t*Bu** (red) and I-C$_6$F$_4$-I (blue).

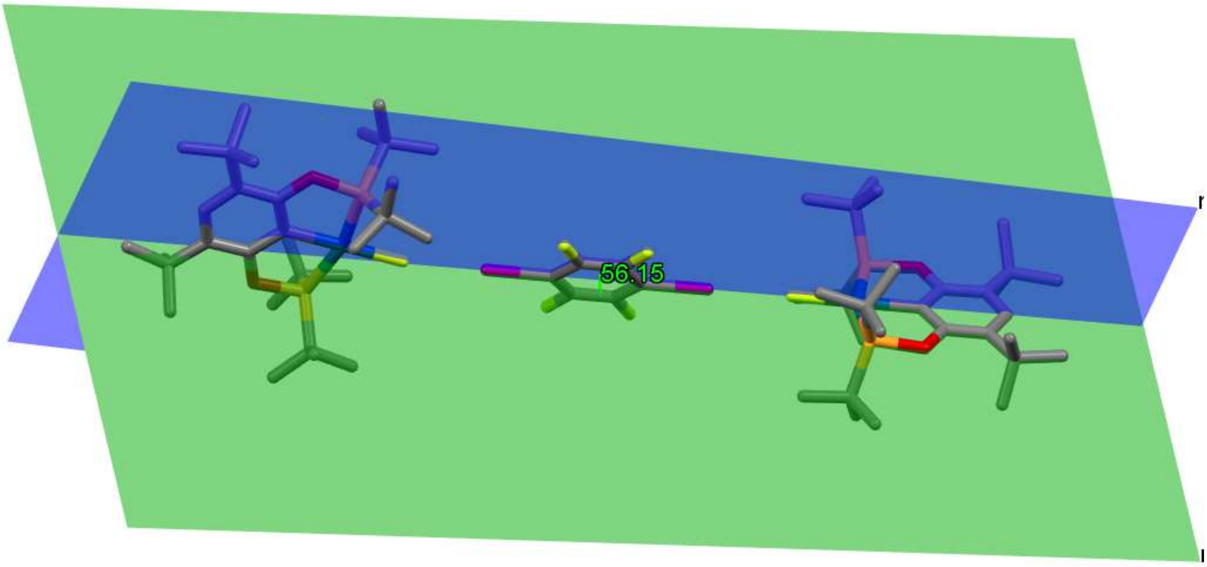


**Figure S15c.** DFT optimised structure of **2-*t*Bu·I-C$_6$F$_4$-I** in toluene, depicting the planes (coloured green and blue) passing through the benzene ring of one of the two molecules of **2-*t*Bu** (green) and I-C$_6$F$_4$-I (blue).

**Table S9a.** Selected experimental (SC-XRD) and density functional theory (DFT) calculated {6-31G* [for C, H, O], 6-31+G* [for F], LANL2DZ [for Pd, P, I]} bond distances (Å) and angles (°) in **2-*t*Bu·I-$C_6F_4$-I** in gas phase.

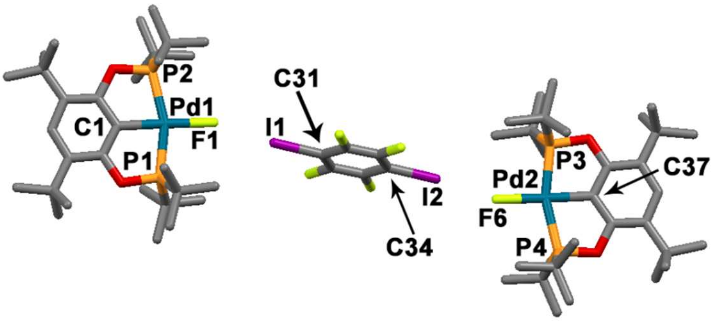


| | X-ray (experimental) | DFT Calculated (gas phase) | | | |
|---|---|---|---|---|---|
| | | B3PW91 | BHandHLYP | M06 | B3LYP |
| Pd1-C1 | 1.998(3) | 2.0078 | 2.0107 | 2.0243 | 2.0225 |
| Pd1-P1 | 2.2723(7) | 2.3354 | 2.3561 | 2.3524 | 2.3632 |
| Pd1-P2 | 2.2852(7) | 2.3375 | 2.3487 | 2.3323 | 2.3552 |
| **Pd1-F1** | **2.0681(17)** | **2.0755** | **2.0577** | **2.0958** | **2.0847** |
| **F1·····I1** | **2.6828(18)** | **2.6763** | **2.6698** | **2.6003** | **2.7020** |
| **I1-C31** | **2.115(3)** | **2.1136** | **2.1041** | **2.1155** | **2.1231** |
| P1-Pd1-C1 | 80.39(8) | 81.97 | 81.51 | 81.28 | 82.01 |
| P1-Pd1-P2 | 160.91(3) | 163.99 | 163.07 | 162.61 | 163.97 |
| P1-Pd1-F1 | 98.36(5) | 97.41 | 99.57 | 103.83 | 99.18 |
| C1-Pd1-P2 | 80.53(8) | 82.01 | 81.56 | 81.35 | 81.99 |
| C1-Pd1-F1 | 178.74(9) | 179.25 | 178.86 | 173.30 | 178.41 |
| P2-Pd1-F1 | 100.72(5) | 98.60 | 97.35 | 93.55 | 96.83 |
| **Pd1-F1·····I1** | **160.03(9)** | **176.50** | **167.80** | **147.19** | **166.34** |
| **F1·····I1-C31** | **174.38(8)** | **179.43** | **178.66** | **178.68** | **178.42** |
| Pd2-C37 | 1.998(3) | 2.0085 | 2.0107 | 2.0261 | 2.0212 |
| Pd2-P3 | 2.2723(7) | 2.3367 | 2.3507 | 2.3571 | 2.3593 |
| Pd2-P4 | 2.2852(7) | 2.3368 | 2.3511 | 2.3329 | 2.3571 |
| **Pd2-F6** | **2.0681(17)** | **2.0740** | **2.0589** | **2.0261** | **2.0888** |
| **F6·····I2** | **2.6828(18)** | **2.6766** | **2.6746** | **2.6158** | **2.7068** |
| **I2-C34** | **2.115(3)** | **2.1137** | **2.1040** | **2.1161** | **2.1229** |
| P3-Pd2-C37 | 80.39(8) | 81.94 | 81.55 | 81.29 | 82.02 |
| P3-Pd2-P4 | 160.91(3) | 163.92 | 163.06 | 161.97 | 164.02 |
| P3-Pd2-F6 | 98.36(5) | 98.53 | 98.49 | 103.11 | 98.75 |
| C37-Pd2-P4 | 80.53(8) | 81.98 | 81.54 | 81.12 | 82.01 |
| C37-Pd2-F6 | 178.74(9) | 179.42 | 179.94 | 173.40 | 179.21 |
| P4-Pd2-F6 | 100.72(5) | 97.55 | 98.41 | 94.76 | 97.21 |
| **Pd2-F6·····I2** | **160.03(9)** | **174.10** | **179.45** | **143.20** | **178.59** |
| **F6·····I2-C34** | **174.38(8)** | **179.41** | **179.64** | **176.18** | **179.64** |
| **RMSE[a]** | | **3.0691** | **3.0407** | **3.8085** | **3.1092** |

| | X-ray (experimental) | DFT Calculated (gas phase) | | | |
|---|---|---|---|---|---|
| | | B3PW91 | BHandHLYP | M06 | B3LYP |
| Pd1-F1 | 2.0681(17) | 2.0755 | 2.0577 | 2.0958 | 2.0847 |
| F1·····I1 | 2.6828(18) | 2.6763 | 2.6698 | 2.6003 | 2.7020 |
| I1-C31 | 2.115(3) | 2.1136 | 2.1041 | 2.1155 | 2.1231 |

| | | | | | |
|---|---|---|---|---|---|
| Pd1-F1·····I1 | 160.03(9) | 176.50 | 167.80 | 147.19 | 166.34 |
| F1·····I1-C31 | 174.38(8) | 179.43 | 178.66 | 178.68 | 178.42 |
| Pd2-F6 | 2.0681(17) | 2.0740 | 2.0589 | 2.0261 | 2.0888 |
| F6·····I2 | 2.6828(18) | 2.6766 | 2.6746 | 2.6158 | 2.7068 |
| I2-C34 | 2.115(3) | 2.1137 | 2.1040 | 2.1161 | 2.1229 |
| Pd2-F6·····I2 | 160.03(9) | 174.10 | 179.45 | 143.20 | 178.59 |
| F6·····I2-C34 | 174.38(8) | 179.41 | 179.64 | 176.18 | 179.64 |
| **RMSE[a]** | | **4.4815** | **4.3328** | **4.5119** | **4.1008** |

[a] **RMSE** = root mean square error between the DFT calculated and experimental bond distances and angles.

**Table S9b.** Selected experimental (SC-XRD) and density functional theory (DFT) calculated {6-31G* [for C, H, O], 6-31+G* [for F], LANL2DZ [for Pd, P, I]} bond distances (Å) and angles (°) in **2-*t*Bu·I-$C_6F_4$-I** in toluene (CPCM).

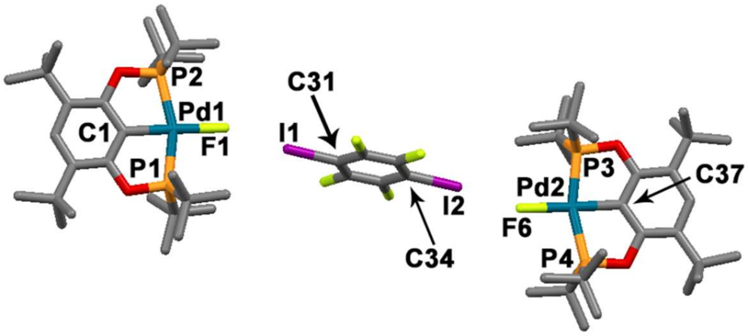


| | X-ray (experimental) | DFT Calculated (toluene) |
|---|---|---|
| | | **B3LYP** |
| Pd1-C1 | 1.998(3) | 2.0228 |
| Pd1-P1 | 2.2723(7) | 2.3726 |
| Pd1-P2 | 2.2852(7) | 2.3643 |
| **Pd1-F1** | **2.0681(17)** | **2.1074** |
| **F1·····I1** | **2.6828(18)** | **2.7035** |
| **I1-C31** | **2.115(3)** | **2.1243** |
| P1-Pd1-C1 | 80.39(8) | 81.90 |
| P1-Pd1-P2 | 160.91(3) | 163.79 |
| P1-Pd1-F1 | 98.36(5) | 99.48 |
| C1-Pd1-P2 | 80.53(8) | 81.89 |
| C1-Pd1-F1 | 178.74(9) | 178.34 |
| P2-Pd1-F1 | 100.72(5) | 96.73 |
| **Pd1-F1·····I1** | **160.03(9)** | **163.24** |
| **F1·····I1-C31** | **174.38(8)** | **179.17** |
| Pd2-C37 | 1.998(3) | 2.0227 |
| Pd2-P3 | 2.2723(7) | 2.3698 |
| Pd2-P4 | 2.2852(7) | 2.3669 |
| **Pd2-F6** | **2.0681(17)** | **2.1091** |
| **F6·····I2** | **2.6828(18)** | **2.7064** |
| **I2-C34** | **2.115(3)** | **2.1238** |
| P3-Pd2-C37 | 80.39(8) | 81.93 |
| P3-Pd2-P4 | 160.91(3) | 163.83 |
| P3-Pd2-F6 | 98.36(5) | 98.92 |
| C37-Pd2-P4 | 80.53(8) | 81.90 |
| C37-Pd2-F6 | 178.74(9) | 179.13 |
| P4-Pd2-F6 | 100.72(5) | 97.24 |
| **Pd2-F6·····I2** | **160.03(9)** | **178.99** |
| **F6·····I2-C34** | **174.38(8)** | **179.45** |
| **RMSE[a]** | | **3.1858** |

[a] **RMSE** = root mean square error between the DFT calculated and experimental bond distances and angles.

**Table S10a.** Selected experimental (SC-XRD) and density functional theory (DFT) calculated {6-31G* [for C, H, O], 6-31+G* [for F], LANL2DZ [for Pd, P]} bond distances (Å) and angles (°) in **2-*t*Bu** in gas phase.

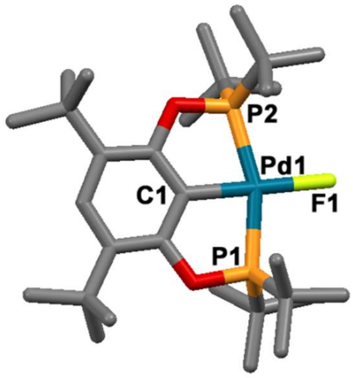


| | X-ray (experimental) | DFT Calculated (gas phase) | | | |
|---|---|---|---|---|---|
| | | **B3PW91** | **BHandHLYP** | **M06** | **B3LYP** |
| Pd1-C1 | 1.979(2) | 2.0122 | 2.0141 | 2.0299 | 2.0251 |
| Pd1-P1 | 2.2666(5) | 2.3323 | 2.3472 | 2.3373 | 2.3534 |
| Pd1-P2 | 2.2675(12)<br>2.2675(13)[a] | 2.3296 | 2.3448 | 2.3368 | 2.3531 |
| Pd1-F1 | **2.0304(16)** | 2.0518 | **2.0374** | **2.0670** | 2.0638 |
| P1-Pd1-C1 | 80.75(6) | 81.91 | 81.48 | 81.04 | 81.91 |
| P1-Pd1-P2 | 161.27(4)<br>161.18(5)[a] | 163.79 | 162.96 | 162.02 | 163.87 |
| P1-Pd1-F1 | 98.67(5) | 98.82 | 98.93 | 99.42 | 98.07 |
| C1-Pd1-P2 | 80.78(7)<br>80.50(7)[a] | 81.88 | 81.48 | 80.99 | 81.98 |
| C1-Pd1-F1 | 179.23(8) | 179.25 | 179.51 | 179.52 | 179.89 |
| P2-Pd1-F1 | 99.82(6)<br>100.07(6)[a] | 97.39 | 98.11 | 98.55 | 98.03 |
| **RMSE[b]** | | **1.7927** | **1.8228** | **1.7768** | **2.1826** |

[a]Two values are given due to disorder over two sites. [b]**RMSE** = root mean square error between the DFT calculated and experimental bond distances and angles; the experimental value closer to the DFT calculated value was chosen for RMSE calculations.

**Table S10b.** Selected experimental (SC-XRD) and density functional theory (DFT) calculated {6-31G* [for C, H, O], 6-31+G* [for F], LANL2DZ [for Pd, P]} bond distances (Å) and angles (°) in **2-*t*Bu** in toluene (CPCM).

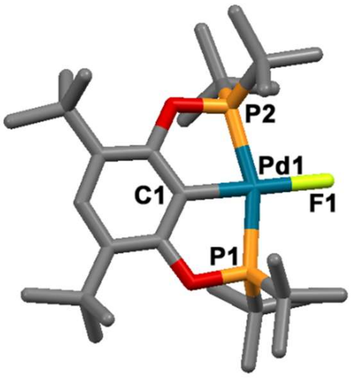


| | X-ray (experimental) | DFT Calculated (toluene) | | | |
|---|---|---|---|---|---|
| | | **B3PW91** | **BHandHLYP** | **M06** | **B3LYP** |
| Pd1-C1 | 1.979(2) | 2.0126 | 2.0148 | 2.0304 | 2.0255 |
| Pd1-P1 | 2.2666(5) | 2.3429 | 2.3586 | 2.3462 | 2.3658 |
| Pd1-P2 | 2.2675(12)<br>2.2675(13)[a] | 2.3416 | 2.3575 | 2.3463 | 2.3650 |
| Pd1-F1 | **2.0304(16)** | 2.0720 | **2.0600** | **2.0926** | 2.0866 |
| P1-Pd1-C1 | 80.75(6) | 81.84 | 81.37 | 80.96 | 81.83 |
| P1-Pd1-P2 | 161.27(4)<br>161.18(5)[a] | 163.65 | 162.73 | 161.86 | 163.72 |
| P1-Pd1-F1 | 98.67(5) | 98.70 | 98.96 | 99.41 | 98.22 |
| C1-Pd1-P2 | 80.78(7)<br>80.50(7)[a] | 81.81 | 81.36 | 80.90 | 81.91 |
| C1-Pd1-F1 | 179.23(8) | 179.45 | 179.66 | 179.61 | 179.93 |
| P2-Pd1-F1 | 99.82(6)<br>100.07(6)[a] | 97.65 | 98.31 | 98.73 | 98.03 |
| **RMSE[a]** | | **1.9864** | **2.0490** | **2.0631** | **2.4588** |

[a]Two values are given due to disorder over two sites. [b]**RMSE** = root mean square error between the DFT calculated and experimental bond distances and angles; the experimental value closer to the DFT calculated value was chosen for RMSE calculations.

**Table S11a.** Selected experimental (SC-XRD) and density functional theory (DFT) calculated {6-31G* [for C, H, O], 6-31+G* [for F], LANL2DZ [for Ni, P]; gas phase} bond distances (Å) and angles (°) in **1-*t*Bu**.

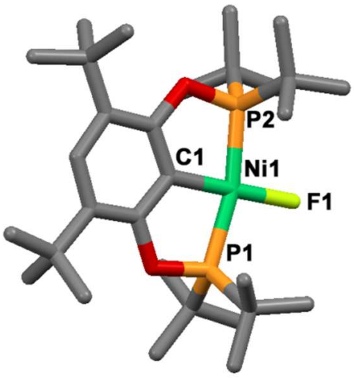


| | X-ray (experimental)[a] | DFT Calculated (gas phase) | | | |
|---|---|---|---|---|---|
| | | B3PW91 | BHandHLYP | M06 | B3LYP |
| Ni1-C1 | 1.8894(19)<br>1.8898(19) | 1.9081 | 1.9256 | 1.9064 | 1.9188 |
| Ni1-P1 | 2.1556(6)<br>2.1526(6) | 2.2137 | 2.2580 | 2.1934 | 2.2345 |
| Ni1-P2 | 2.1628(6)<br>2.1560(6) | 2.2137 | 2.2569 | 2.1917 | 2.2335 |
| Ni1-F1 | **1.8417(13)**<br>**1.8418(12)** | 1.8549 | **1.8246** | **1.8630** | 1.8614 |
| P1-Ni1-C1 | 82.51(6)<br>82.88(6) | 83.92 | 83.30 | 83.56 | 84.06 |
| P1-Ni1-P2 | 165.30(3)<br>165.06(2) | 167.81 | 166.59 | 167.05 | 168.06 |
| P1-Ni1-F1 | 96.77(5)<br>96.71(4) | 96.07 | 96.78 | 96.56 | 96.02 |
| C1-Ni1-P2 | 82.89(6)<br>82.48(6) | 83.90 | 83.31 | 83.50 | 84.02 |
| C1-Ni1-F1 | 178.83(9)<br>179.52(8) | 179.41 | 179.64 | 179.43 | 179.92 |
| P2-Ni1-F1 | 97.85(5)<br>97.94(4) | 96.11 | 96.60 | 96.36 | 95.90 |
| **RMSE[a]** | | **1.5237** | **2.2073** | **1.0788** | **1.9691** |

[a]Two values given due to two molecules in the asymmetric unit. [b]**RMSE** = root mean square error between the DFT calculated and experimental bond distances and angles; the experimental value closer to the DFT calculated value was chosen for RMSE calculations.

**Table S11b.** Selected experimental (SC-XRD) and density functional theory (DFT) calculated {6-31G* [for C, H, O], 6-31+G* [for F], LANL2DZ [for Ni, P]; toluene, CPCM} bond distances (Å) and angles (°) in **1-*t*Bu**.

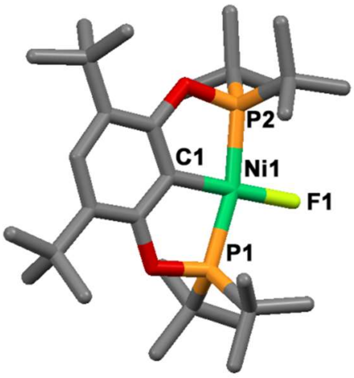


| | X-ray (experimental)[a] | DFT Calculated (toluene) | | | |
|---|---|---|---|---|---|
| | | B3PW91 | BHandHLYP | M06 | B3LYP |
| Ni1-C1 | 1.8894(19)<br>1.8898(19) | 1.9097 | 1.9284 | 1.9086 | 1.9205 |
| Ni1-P1 | 2.1556(6)<br>2.1526(6) | 2.2245 | 2.2713 | 2.2060 | 2.2459 |
| Ni1-P2 | 2.1628(6)<br>2.1560(6) | 2.2235 | 2.2696 | 2.2003 | 2.2444 |
| Ni1-F1 | **1.8417(13)**<br>**1.8418(12)** | 1.8653 | **1.8363** | **1.8736** | 1.8730 |
| P1-Ni1-C1 | 82.51(6)<br>82.88(6) | 83.86 | 83.20 | 83.52 | 83.98 |
| P1-Ni1-P2 | 165.30(3)<br>165.06(2) | 167.70 | 166.40 | 166.91 | 167.90 |
| P1-Ni1-F1 | 96.77(5)<br>96.71(4) | 96.19 | 96.89 | 96.99 | 96.12 |
| C1-Ni1-P2 | 82.89(6)<br>82.48(6) | 83.84 | 83.23 | 83.39 | 83.96 |
| C1-Ni1-F1 | 178.83(9)<br>179.52(8) | 179.50 | 179.74 | 178.69 | 179.82 |
| P2-Ni1-F1 | 97.85(5)<br>97.94(4) | 96.11 | 96.68 | 96.08 | 95.94 |
| **RMSE[a]** | | **1.7059** | **2.4432** | **1.3387** | **2.1772** |

[a]Two values given due to two molecules in the asymmetric unit. [b]**RMSE** = root mean square error between the DFT calculated and experimental bond distances and angles; the experimental value closer to the DFT calculated value was chosen for RMSE calculations.

**Table S12a.** Selected experimental (SC-XRD) and density functional theory (DFT) calculated {6-31G* [for C, H, O], 6-31+G* [for F], LANL2DZ [for Pt, P]; gas phase} bond distances (Å) and angles (°) in **3-*t*Bu**.

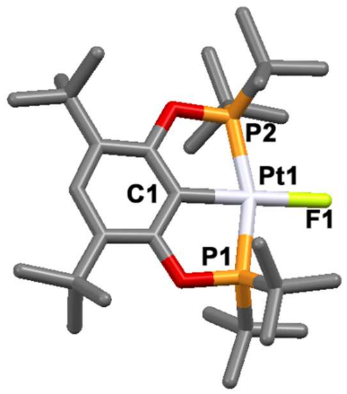


| | **X-ray (experimental)** | **DFT Calculated (gas phase)** | | | |
|---|---|---|---|---|---|
| | | **B3PW91** | **BHandHLYP** | **M06** | **B3LYP** |
| Pt1-C1 | 1.981(2) | 2.0036 | 2.0067 | 2.0186 | 2.0161 |
| Pt1-P1 | 2.2618(6) | 2.3301 | 2.3402 | 2.3405 | 2.3485 |
| Pt1-P2 | 2.2607(6) | 2.3292 | 2.3402 | 2.3408 | 2.3483 |
| Pt1-F1 | **2.0539(15)** | 2.0742 | **2.0590** | **2.0872** | 2.0841 |
| P1-Pt1-C1 | 81.16(6) | 82.23 | 81.84 | 81.37 | 82.25 |
| P1-Pt1-P2 | 162.00(2) | 164.46 | 163.66 | 162.71 | 164.47 |
| P1-Pt1-F1 | 98.50(4) | 98.41 | 98.15 | 99.04 | 97.80 |
| C1-Pt1-P2 | 80.85(6) | 82.23 | 81.82 | 81.34 | 82.23 |
| C1-Pt1-F1 | 179.56(8) | 179.34 | 179.36 | 179.59 | 179.73 |
| P2-Pt1-F1 | 99.48(4) | 97.12 | 98.17 | 98.25 | 97.73 |
| **RMSE[a]** | | **1.8217** | **1.7661** | **1.8261** | **2.1368** |

[a] **RMSE** = root mean square error between the DFT calculated and experimental bond distances and angles.

**Table S12b.** Selected experimental (SC-XRD) and density functional theory (DFT) calculated {6-31G* [for C, H, O], 6-31+G* [for F], LANL2DZ [for Pt, P]; toluene, CPCM} bond distances (Å) and angles (°) in **3-*t*Bu**.

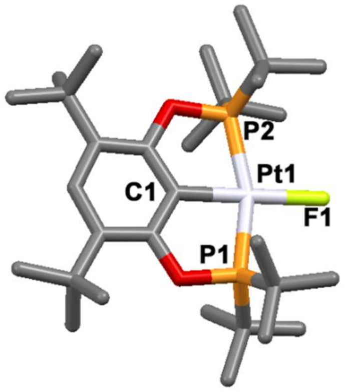


| | X-ray (experimental) | DFT Calculated (toluene) | | | |
|---|---|---|---|---|---|
| | | **B3PW91** | **BHandHLYP** | **M06** | **B3LYP** |
| Pt1-C1 | 1.981(2) | 2.0033 | 2.0069 | 2.0184 | 2.0162 |
| Pt1-P1 | 2.2618(6) | 2.3394 | 2.3501 | 2.3540 | 2.3595 |
| Pt1-P2 | 2.2607(6) | 2.3409 | 2.3513 | 2.3512 | 2.3593 |
| Pt1-F1 | **2.0539(15)** | 2.0929 | **2.0799** | **2.1108** | 2.1042 |
| P1-Pt1-C1 | 81.16(6) | 82.20 | 81.78 | 81.29 | 82.21 |
| P1-Pt1-P2 | 162.00(2) | 164.37 | 163.49 | 162.61 | 164.40 |
| P1-Pt1-F1 | 98.50(4) | 98.33 | 98.36 | 100.06 | 97.84 |
| C1-Pt1-P2 | 80.85(6) | 82.17 | 81.73 | 81.32 | 82.20 |
| C1-Pt1-F1 | 179.56(8) | 179.47 | 179.80 | 178.52 | 179.67 |
| P2-Pt1-F1 | 99.48(4) | 97.30 | 98.13 | 97.33 | 97.75 |
| **RMSE[a]** | | **2.0127** | **1.9776** | **2.2782** | **2.3868** |

[a]**RMSE** = root mean square error between the DFT calculated and experimental bond distances and angles

**Table S13.** Selected density functional theory (DFT) calculated {6-31G* [for C, H, O], 6-31+G* [for F], LANL2DZ [for Ni, P, I]} bond distances (Å) and angles (°) in **1-*t*Bu·$C_6F_5$I**.

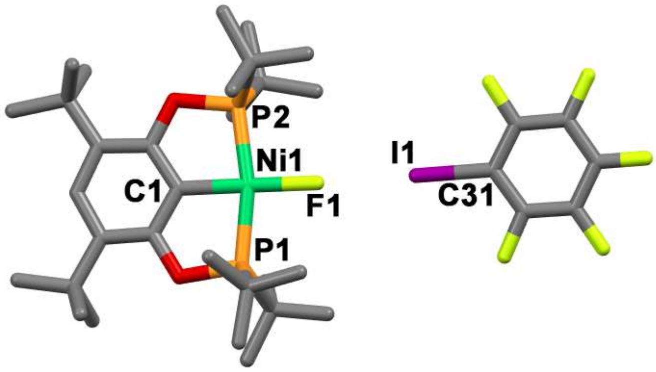


| | gas phase | | | |
|---|---|---|---|---|
| | **B3PW91** | **BHandHLYP** | **M06** | **B3LYP** |
| Ni1-C1 | 1.9037 | 1.9194 | 1.8982 | 1.9153 |
| Ni1-P1 | 2.2227 | 2.2645 | 2.1972 | 2.2434 |
| Ni1-P2 | 2.2243 | 2.2653 | 2.1926 | 2.2445 |
| **Ni1-F1** | **1.8785** | **1.8467** | **1.8828** | **1.8858** |
| **F1·····I1** | **2.6993** | **2.6945** | **2.6261** | **2.7175** |
| **I1-C31** | **2.1127** | **2.1029** | **2.1125** | **2.1220** |
| P1-Ni1-C1 | 83.97 | 83.32 | 83.98 | 84.01 |
| P1-Ni1-P2 | 167.93 | 166.68 | 167.66 | 168.00 |
| P1-Ni1-F1 | 95.67 | 96.54 | 96.78 | 95.73 |
| C1-Ni1-P2 | 83.97 | 83.36 | 83.70 | 84.01 |
| C1-Ni1-F1 | 179.61 | 179.86 | 179.17 | 179.67 |
| P2-Ni1-F1 | 96.38 | 96.77 | 95.55 | 96.25 |
| **Ni1-F1·····I1** | **177.53** | **179.52** | **171.30** | **177.83** |
| **F1·····I1-C31** | **179.68** | **179.63** | **178.77** | **179.79** |

| | toluene | | | |
|---|---|---|---|---|
| | **B3PW91** | **BHandHLYP** | **M06** | **B3LYP** |
| Ni1-C1 | 1.9051 | 1.9227 | 1.8993 | 1.9172 |
| Ni1-P1 | 2.2290 | 2.2726 | 2.2013 | 2.2503 |
| Ni1-P2 | 2.2306 | 2.2741 | 2.1971 | 2.2516 |
| **Ni1-F1** | **1.8847** | **1.8533** | **1.8894** | **1.8929** |
| **F1·····I1** | **2.7077** | **2.7060** | **2.6237** | **2.7290** |
| **I1-C31** | **2.1132** | **2.1029** | **2.1135** | **2.1222** |
| P1-Ni1-C1 | 83.95 | 83.25 | 83.94 | 83.96 |
| P1-Ni1-P2 | 167.88 | 166.53 | 167.61 | 167.91 |
| P1-Ni1-F1 | 95.76 | 96.61 | 96.80 | 95.85 |
| C1-Ni1-P2 | 83.95 | 83.29 | 83.69 | 83.97 |
| C1-Ni1-F1 | 179.57 | 179.85 | 179.15 | 179.72 |
| P2-Ni1-F1 | 96.34 | 96.85 | 95.58 | 96.23 |
| **Ni1-F1·····I1** | **177.27** | **179.49** | **171.47** | **177.36** |
| **F1·····I1-C31** | **179.59** | **179.53** | **179.11** | **179.63** |

**Table S14**. Selected density functional theory (DFT) calculated {6-31G* [for C, H, O], 6-31+G* [for F], LANL2DZ [for Pd, P, I]} bond distances (Å) and angles (°) in **2-*t*Bu·$C_6F_5$I**.

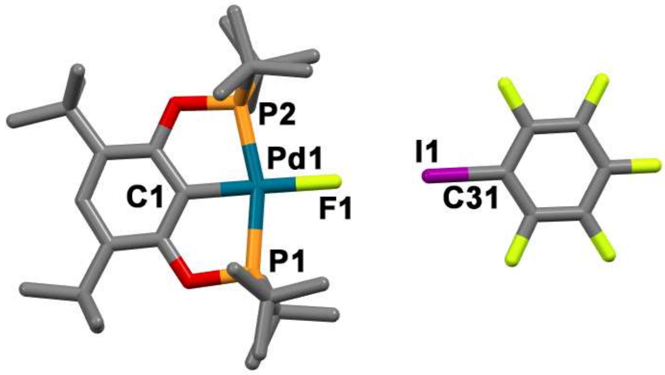


| | gas phase | | | |
|---|---|---|---|---|
| | **B3PW91** | **BHandHLYP** | **M06** | **B3LYP** |
| Pd1-C1 | 2.0085 | 2.0100 | 2.0229 | 2.0220 |
| Pd1-P1 | 2.3383 | 2.3545 | 2.3442 | 2.3565 |
| Pd1-P2 | 2.3418 | 2.3524 | 2.3411 | 2.3652 |
| **Pd1-F1** | **2.0793** | **2.0635** | **2.0938** | **2.0941** |
| **F1·····I1** | **2.6299** | **2.6219** | **2.5684** | **2.6485** |
| **I1-C31** | **2.1183** | **2.1085** | **2.1183** | **2.1279** |
| P1- Pd1-C1 | 82.00 | 81.47 | 81.31 | 81.91 |
| P1- Pd1-P2 | 163.90 | 162.99 | 162.53 | 163.88 |
| P1- Pd1-F1 | 97.26 | 98.72 | 99.43 | 96.25 |
| C1- Pd1-P2 | 82.011 | 81.52 | 81.34 | 81.97 |
| C1- Pd1-F1 | 178.96 | 179.44 | 178.52 | 177.71 |
| P2- Pd1-F1 | 98.70 | 98.29 | 97.97 | 99.85 |
| **Pd1-F1·····I1** | **163.39** | **169.21** | **167.77** | **163.39** |
| **F1·····I1-C31** | **179.47** | **179.54** | **179.40** | **179.75** |

| | toluene | | | |
|---|---|---|---|---|
| | **B3PW91** | **BHandHLYP** | **M06** | **B3LYP** |
| Pd1-C1 | 2.0093 | 2.0116 | 2.0245 | 2.0235 |
| Pd1-P1 | 2.3447 | 2.3599 | 2.3489 | 2.3657 |
| Pd1-P2 | 2.3492 | 2.3628 | 2.3456 | 2.3729 |
| **Pd1-F1** | **2.0950** | **2.0797** | **2.1119** | **2.1113** |
| **F1·····I1** | **2.6421** | **2.6408** | **2.5820** | **2.6628** |
| **I1-C31** | **2.1186** | **2.1080** | **2.1194** | **2.1283** |
| P1- Pd1-C1 | 81.94 | 81.43 | 81.10 | 81.84 |
| P1- Pd1-P2 | 163.74 | 162.86 | 161.87 | 163.73 |
| P1- Pd1-F1 | 97.10 | 97.94 | 100.49 | 96.77 |
| C1- Pd1-P2 | 81.95 | 81.43 | 81.32 | 81.89 |
| C1- Pd1-F1 | 178.93 | 179.15 | 175.02 | 178.03 |
| P2- Pd1-F1 | 99.02 | 99.20 | 97.40 | 99.49 |
| **Pd1-F1·····I1** | **165.86** | **164.47** | **150.21** | **160.30** |
| **F1·····I1-C31** | **179.43** | **179.72** | **178.33** | **179.34** |

**Table S15.** Selected density functional theory (DFT) calculated {6-31G* [for C, H, O], 6-31+G* [for F], LANL2DZ [for Pt, P, I]} bond distances (Å) and angles (°) in **3-*t*Bu·$C_6F_5$I**.

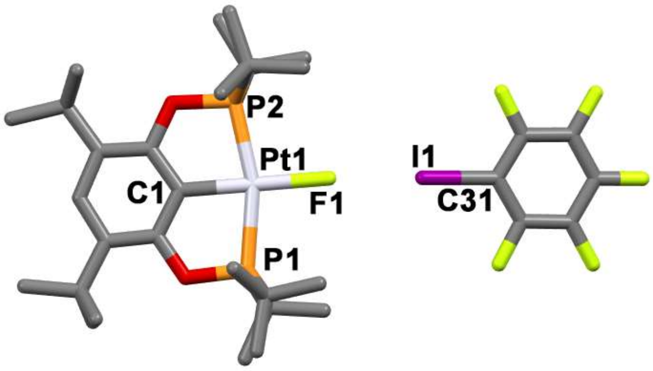


| | gas phase | | | |
|---|---|---|---|---|
| | **B3PW91** | **BHandHLYP** | **M06** | **B3LYP** |
| Pt1-C1 | 2.0000 | 2.0036 | 2.0121 | 2.0127 |
| Pt1-P1 | 2.3362 | 2.3475 | 2.3441 | 2.3560 |
| Pt1-P2 | 2.3392 | 2.3476 | 2.3488 | 2.3575 |
| **Pt1-F1** | **2.0975** | **2.0801** | **2.1122** | **2.1124** |
| **F1·····I1** | **2.6618** | **2.6456** | **2.5977** | **2.6660** |
| **I1-C31** | **2.1148** | **2.1058** | **2.1146** | **2.1251** |
| P1- Pt1-C1 | 82.34 | 81.84 | 81.57 | 82.29 |
| P1- Pt1-P2 | 164.57 | 163.66 | 163.19 | 164.52 |
| P1- Pt1-F1 | 97.00 | 98.36 | 98.04 | 97.67 |
| C1- Pt1-P2 | 82.32 | 81.86 | 81.68 | 82.29 |
| C1- Pt1-F1 | 179.10 | 179.05 | 179.48 | 178.67 |
| P2- Pt1-F1 | 98.31 | 97.91 | 98.72 | 97.72 |
| **Pt1-F1·····I1** | **164.59** | **162.43** | **167.70** | **159.99** |
| **F1·····I1-C31** | **179.63** | **179.57** | **179.03** | **179.09** |

| | toluene | | | |
|---|---|---|---|---|
| | **B3PW91** | **BHandHLYP** | **M06** | **B3LYP** |
| Pt1-C1 | 1.9999 | 2.0044 | 2.0132 | 2.0131 |
| Pt1-P1 | 2.3424 | 2.3540 | 2.3568 | 2.3637 |
| Pt1-P2 | 2.3466 | 2.3546 | 2.3543 | 2.3642 |
| **Pt1-F1** | **2.1132** | **2.0965** | **2.1279** | **2.1300** |
| **F1·····I1** | **2.6748** | **2.6630** | **2.6016** | **2.6828** |
| **I1-C31** | **2.1153** | **2.1054** | **2.1163** | **2.1254** |
| P1- Pt1-C1 | 82.33 | 81.79 | 81.45 | 82.24 |
| P1- Pt1-P2 | 164.57 | 163.54 | 163.03 | 164.43 |
| P1- Pt1-F1 | 96.85 | 98.16 | 100.17 | 97.48 |
| C1- Pt1-P2 | 82.30 | 81.75 | 81.65 | 82.20 |
| C1- Pt1-F1 | 178.92 | 179.13 | 178.37 | 178.86 |
| P2- Pt1-F1 | 98.49 | 98.29 | 96.73 | 98.07 |
| **Pt1-F1·····I1** | **161.81** | **159.66** | **161.14** | **158.17** |
| **F1·····I1-C31** | **179.49** | **179.34** | **177.76** | **178.60** |

**Table S16*o***. Bond distances between the **H**$^{tBu\text{-}CH3}$ on P and *ortho* **F** on $C_6F_5I$ in DFT optimised structure (B3LYP/LANL2DZ/6-31G*/6-31+G*/toluene) of **1-*t*Bu·$C_6F_5$I**.

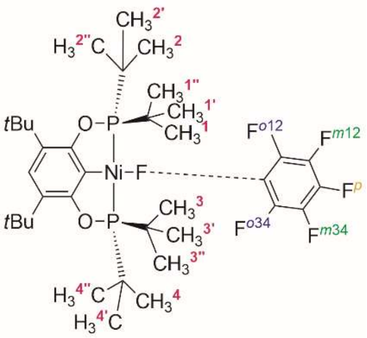


| | H·····F$^{o}$ distance (Å) | Average H·····F$^{o}$ distance (Å) | Average of the shortest H·····F$^{o}$ distances (Å) |
|---|---|---|---|
| H$^{1}$·····F$^{o12}$ | 5.39319 | | **5.2572** < H$^{1}$·····F$^{o12}$, H$^{2}$·····F$^{o12}$, H$^{3'}$·····F$^{o34}$, H$^{4'}$·····F$^{o34}$> |
| | 6.03446 | **5.4411** | |
| | 4.89582 | | |
| H$^{1'}$·····F$^{o12}$ | 8.33315 | | |
| | 7.95144 | 8.4348 | |
| | 9.01972 | | |
| H$^{1''}$·····F$^{o12}$ | 7.96183 | | |
| | 8.52805 | 7.8421 | |
| | 7.03634 | | |
| H$^{2}$·····F$^{o12}$ | 4.51188 | | |
| | 5.17537 | **4.8432** | |
| | 4.84231 | | |
| H$^{2'}$·····F$^{o12}$ | 6.85675 | | |
| | 7.3409 | 7.4945 | |
| | 8.28601 | | |
| H$^{2''}$·····F$^{o12}$ | 7.63323 | | |
| | 7.35508 | 7.8410 | |
| | 8.53486 | | |
| H$^{3}$·····F$^{o34}$ | 7.81058 | | |
| | 7.66881 | 8.0711 | |
| | 8.73406 | | |
| H$^{3'}$·····F$^{o34}$ | 5.27326 | | |
| | 5.13939 | **4.9932** | |
| | 4.56691 | | |
| H$^{3''}$·····F$^{o34}$ | 7.29665 | | |
| | 6.7569 | 7.4424 | |
| | 8.27357 | | |
| H$^{4}$·····F$^{o34}$ | 8.78248 | | |
| | 7.30328 | 8.1216 | |
| | 8.27915 | | |
| H$^{4'}$·····F$^{o34}$ | 5.18931 | | |
| | 6.38728 | **5.7515** | |
| | 5.67805 | | |
| H$^{4''}$·····F$^{o34}$ | 8.63616 | | |
| | 8.2221 | 8.7139 | |
| | 9.28335 | | |

**Table S16*m***. Bond distances between the **H**$^{tBu\text{-}CH3}$ on P and *meta* **F** on $C_6F_5I$ in DFT optimised structure (B3LYP/LANL2DZ/6-31G*/6-31+G*/toluene) of **1-*t*Bu·$C_6F_5$I**.

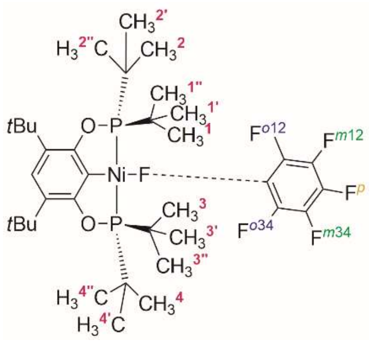


| | H·····$F^m$ distance (Å) | Average H·····$F^m$ distance (Å) | Average of the shortest H·····$F^m$ distances (Å) |
|---|---|---|---|
| $H^{1}$·····$F^{m12}$ | 10.72617 | 10.9162 | **7.7688** <$H^{1'}$·····$F^{m12}$, $H^{2'}$·····$F^{m12}$, $H^{3'}$·····$F^{m34}$, $H^{4'}$·····$F^{m34}$> |
| | 10.44087 | | |
| | 11.58146 | | |
| $H^{1'}$·····$F^{m12}$ | 8.34075 | **7.8786** | |
| | 7.88222 | | |
| | 7.41276 | | |
| $H^{1''}$·····$F^{m12}$ | 10.3394 | 10.3030 | |
| | 11.0719 | | |
| | 9.49768 | | |
| $H^{2}$·····$F^{m12}$ | 10.91064 | 10.0628 | |
| | 9.43446 | | |
| | 9.84337 | | |
| $H^{2'}$·····$F^{m12}$ | 7.17862 | **7.4225** | |
| | 7.42452 | | |
| | 7.66432 | | |
| $H^{2''}$·····$F^{m12}$ | 11.14214 | 10.3777 | |
| | 10.11432 | | |
| | 9.8768 | | |
| $H^{3}$·····$F^{m34}$ | 10.72617 | 10.6292 | |
| | 10.44087 | | |
| | 11.58146 | | |
| $H^{3'}$·····$F^{m34}$ | 8.34075 | **7.5822** | |
| | 7.88222 | | |
| | 7.41276 | | |
| $H^{3''}$·····$F^{m34}$ | 10.3394 | 10.0116 | |
| | 11.0719 | | |
| | 9.49768 | | |
| $H^{4}$·····$F^{m34}$ | 10.91064 | 10.6081 | |
| | 9.43446 | | |
| | 9.84337 | | |
| $H^{4'}$·····$F^{m34}$ | 7.17862 | **8.1921** | |
| | 7.42452 | | |
| | 7.66432 | | |
| $H^{4''}$·····$F^{m34}$ | 11.14214 | 11.2097 | |
| | 10.11432 | | |
| | 9.8768 | | |

**Table S16*p***. Bond distances between the $\mathbf{H}^{t\text{Bu-CH3}}$ on P and *para* **F** on $C_6F_5I$ in DFT optimised structure (B3LYP/LANL2DZ/6-31G*/6-31+G*/toluene) of **1-*t*Bu·$C_6F_5I$**.

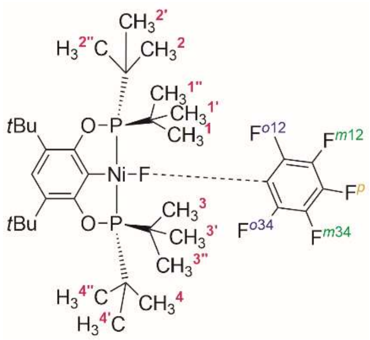


| | H·····$F^p$ distance (Å) | Average H·····$F^p$ distance (Å) | Average of the shortest H·····$F^p$ distances (Å) |
|---|---|---|---|
| $H^{1}$·····$F^{p}$ | 12.1291 | 12.2656 | **9.5363** <$H^{1'}$·····$F^{p}$, $H^{2'}$·····$F^{p}$, $H^{3'}$·····$F^{p}$, $H^{4'}$·····$F^{p}$> |
| | 11.63665 | | |
| | 13.0312 | | |
| $H^{1'}$·····$F^{p}$ | 9.23577 | **9.4550** | |
| | 9.9011 | | |
| | 9.22816 | | |
| $H^{1''}$·····$F^{p}$ | 12.20854 | 12.2526 | |
| | 12.98619 | | |
| | 11.56319 | | |
| $H^{2}$·····$F^{p}$ | 12.0469 | 12.4925 | |
| | 13.1546 | | |
| | 12.27603 | | |
| $H^{2'}$·····$F^{p}$ | 9.52034 | **9.3906** | |
| | 8.92469 | | |
| | 9.72686 | | |
| $H^{2''}$·····$F^{p}$ | 10.93 | 11.7126 | |
| | 12.56082 | | |
| | 11.64697 | | |
| $H^{3}$·····$F^{p}$ | 12.1291 | 12.7093 | |
| | 11.63665 | | |
| | 13.0312 | | |
| $H^{3'}$·····$F^{p}$ | 9.23577 | **9.5632** | |
| | 9.9011 | | |
| | 9.22816 | | |
| $H^{3''}$·····$F^{p}$ | 12.20854 | 11.6464 | |
| | 12.98619 | | |
| | 11.56319 | | |
| $H^{4}$·····$F^{p}$ | 12.0469 | 12.5211 | |
| | 13.1546 | | |
| | 12.27603 | | |
| $H^{4'}$·····$F^{p}$ | 9.52034 | **9.7363** | |
| | 8.92469 | | |
| | 9.72686 | | |
| $H^{4''}$·····$F^{p}$ | 10.93 | 12.5294 | |
| | 12.56082 | | |
| | 11.64697 | | |

**Table S17a**. Bond distances between the $H^{tBu\text{-}CH3}$ on P(1) {and P(2)} and the corresponding F(2) {and F(3)} *ortho* to I(1) on $C_6F_4I_2$ in DFT optimised (B3LYP/LANL2DZ/6-31G*/6-31+G*/toluene) structures of **2-*t*Bu·I-$C_6F_4$-I**.

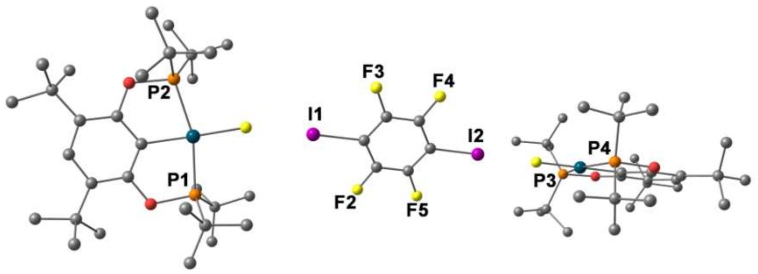


| F | $H^{tBu\text{-}CH3}$-F label | $H^{tBu\text{-}CH3}$-F distance (Å) | Average $H^{tBu\text{-}CH3}$-F distance (Å) |
|---|---|---|---|
| F(2) | F(2)-H(20) | 6.91288 | 6.9678 |
| | F(2)-H(21) | 7.76411 | |
| | F(2)-H(22) | 6.22657 | |
| | F(2)-H(26) | 4.06659 | **4.5574** |
| | F(2)-H(27) | 4.69345 | |
| | F(2)-H(28) | 4.91215 | |
| | F(2)-H(23) | 7.32433 | 7.6224 |
| | F(2)-H(24) | 7.26585 | |
| | F(2)-H(25) | 8.27719 | |
| | | | |
| | F(2)-H(29) | 7.7619 | 7.7850 |
| | F(2)-H(30) | 7.1541 | |
| | F(2)-H(31) | 8.4390 | |
| | F(2)-H(35) | 4.6654 | **4.9765** |
| | F(2)-H(36) | 4.7282 | |
| | F(2)-H(37) | 5.5359 | |
| | F(2)-H(32) | 7.7384 | 7.7841 |
| | F(2)-H(33) | 8.4536 | |
| | F(2)-H(34) | 7.1603 | |
| | | | |
| F(3) | F(3)-H(38) | 8.7772 | 8.6927 |
| | F(3)-H(39) | 9.4470 | |
| | F(3)-H(40) | 7.8540 | |
| | F(3)-H(44) | 6.9673 | **6.4674** |
| | F(3)-H(45) | 5.8416 | |
| | F(3)-H(46) | 6.5932 | |
| | F(3)-H(41) | 9.1160 | 9.5490 |
| | F(3)-H(42) | 10.0951 | |
| | F(3)-H(43) | 9.4358 | |
| | | | |
| | F(3)-H(53) | 8.3530 | 8.9816 |
| | F(3)-H(54) | 9.7171 | |
| | F(3)-H(55) | 8.8748 | |
| | F(3)-H(50) | 5.9981 | **6.2065** |
| | F(3)-H(51) | 5.9170 | |
| | F(3)-H(52) | 6.7044 | |
| | F(3)-H(47) | 9.0302 | 9.0613 |
| | F(3)-H(48) | 9.7408 | |
| | F(3)-H(49) | 8.4128 | |

**Table S17b**. Bond distances between the $H^{tBu\text{-}CH3}$ on P(3) {and P(4)} and F {F(4) and F(5)} *ortho* to I(2) on $C_6F_4I_2$ in DFT optimised (B3LYP/LANL2DZ/6-31G*/6-31+G*/toluene) structures of **2-*t*Bu·I-$C_6F_4$-I**.

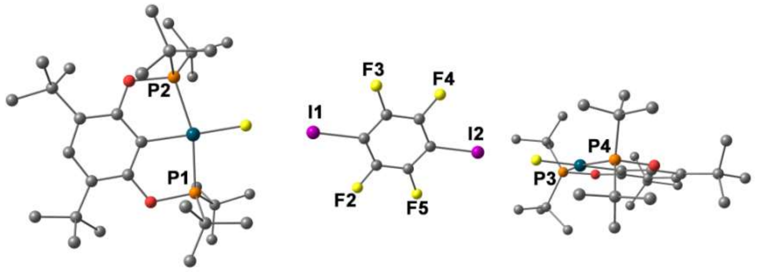


| F | $H^{tBu\text{-}CH3}$-F label | $H^{tBu\text{-}CH3}$-F distance (Å) | Average $H^{tBu\text{-}CH3}$-F distance (Å) |
|---|---|---|---|
| F(4) | F(4)-H(81) | 7.7349 | **7.1470** |
| | F(4)-H(82) | 6.3141 | |
| | F(4)-H(83) | 7.3921 | |
| | F(4)-H(78) | 8.4044 | 8.2580 |
| | F(4)-H(79) | 7.2985 | |
| | F(4)-H(80) | 9.0713 | |
| | F(4)-H(75) | 9.8846 | 10.1671 |
| | F(4)-H(76) | 10.5459 | |
| | F(4)-H(77) | 10.0708 | |
| | | | |
| | F(4)-H(84) | 10.8329 | 10.62781 |
| | F(4)-H(85) | 10.0051 | |
| | F(4)-H(86) | 11.0454 | |
| | F(4)-H(90) | 7.3269 | **7.9217** |
| | F(4)-H(91) | 7.7281 | |
| | F(4)-H(92) | 8.7102 | |
| | F(4)-H(87) | 10.4046 | 10.1635 |
| | F(4)-H(88) | 10.7445 | |
| | F(4)-H(89) | 9.3413 | |
| | | | |
| | F(4)-H(93) | 7.7609 | 7.9788 |
| | F(4)-H(94) | 8.8265 | |
| | F(4)-H(95) | 7.3491 | |
| | F(4)-H(99) | 5.6958 | **5.4429** |
| | F(4)-H(100) | 5.1068 | |
| | F(4)-H(101) | 5.5261 | |
| | F(4)-H(96) | 8.0054 | 8.4783 |
| | F(4)-H(97) | 9.1823 | |
| | F(4)-H(98) | 8.2474 | |
| | | | |
| | F(4)-H(108) | 8.4307 | 9.1516 |
| | F(4)-H(109) | 9.7673 | |
| | F(4)-H(110) | 9.2567 | |
| | F(4)-H(105) | 6.3077 | **6.5478** |
| | F(4)-H(106) | 6.0905 | |
| | F(4)-H(107) | 7.2452 | |
| | F(4)-H(102) | 9.4220 | 9.2928 |
| | F(4)-H(103) | 9.8348 | |
| | F(4)-H(104) | 8.6218 | |
| | | | |
| F(5) | F(5)-H(81) | 6.4746 | **6.5504** |
| | F(5)-H(82) | 5.9580 | |

| | | | |
|---|---|---|---|
| | F(5)-H(83) | 7.2186 | |
| | F(5)-H(78) | 9.1039 | 8.9274 |
| | F(5)-H(79) | 8.1148 | |
| | F(5)-H(80) | 9.5634 | |
| | F(5)-H(75) | 9.4999 | 9.4425 |
| | F(5)-H(76) | 9.9493 | |
| | F(5)-H(77) | 8.8782 | |
| | | | |
| | F(5)-H(84) | 8.2240 | 8.4180 |
| | F(5)-H(85) | 7.8786 | |
| | F(5)-H(86) | 9.1513 | |
| | F(5)-H(90) | 5.2229 | **5.4715** |
| | F(5)-H(91) | 5.4138 | |
| | F(5)-H(92) | 5.7778 | |
| | F(5)-H(87) | 7.9359 | 8.1747 |
| | F(5)-H(88) | 8.9899 | |
| | F(5)-H(89) | 7.5983 | |
| | | | |
| | F(5)-H(93) | 10.2166 | 9.9456 |
| | F(5)-H(94) | 10.5423 | |
| | F(5)-H(95) | 9.0780 | |
| | F(5)-H(99) | 8.5920 | **7.8401** |
| | F(5)-H(100) | 7.1542 | |
| | F(5)-H(101) | 7.7741 | |
| | F(5)-H(96) | 10.0652 | 10.6291 |
| | F(5)-H(97) | 11.0179 | |
| | F(5)-H(98) | 10.8041 | |
| | | | |
| | F(5)-H(108) | 7.5482 | 8.4410 |
| | F(5)-H(109) | 9.2943 | |
| | F(5)-H(110) | 8.4803 | |
| | F(5)-H(105) | 7.3326 | **6.8691** |
| | F(5)-H(106) | 6.1297 | |
| | F(5)-H(107) | 7.1451 | |
| | F(5)-H(102) | 9.7394 | 9.9934 |
| | F(5)-H(103) | 10.4692 | |
| | F(5)-H(104) | 9.7716 | |

## References


1 M. Joksch, J. Haak, A. Spannenberg, T. Beweries, *Eur. J. Inorg. Chem.* **2017**, 3815.

2 M. Joksch, H. Agarwala, J. Haak, A. Spannenberg, T. Beweries, *Polyhedron* **2018**, *143*, 118.

3 G. M. Sheldrick, *Acta Crystallogr.* **2008**, *A64*, 112.

4 G. M. Sheldrick, *Acta Crystallogr.* **2015**, *C71*, 3.

5 Diamond - Crystal and Molecular Structure Visualisation, Crystal Impact - Dr. H. Putz & Dr. K. Brandenburg GbR, Kreuzherrenstr. 102, 53227 Bonn, Germany, http://www.crystalimpact.com/diamond.

6 C. F. Macrae, I. J. Bruno, J. A. Chisholm, P. R. Edgington, P. McCabe, E. Pidcock, L. Rodriguez-Monge, R. Taylor, J. van de Streek and P. A. Wood, *J. Appl. Cryst.* **2008**, *41*, 466.

7 J. S. Renny, L. L. Tomasevich, E. H. Tallmadge, D. B. Collum, *Angew. Chem. Int. Ed.* **2013**, *52*, 11998.

8 M. Findeisen, T. Brand, S. Berger, *Magn. Res. Chem.* **2007**, *45*, 175.

9 M. J. Frisch, G. W. Trucks, H. B. Schlegel, G. E. Scuseria, M. A. Robb, J. R. Cheeseman, G. Scalmani, V. Barone, B. Mennucci, G. A. Petersson, H. Nakatsuji, M. Caricato, X. Li, H. P. Hratchian, A. F. Izmaylov, J. Bloino, G. Zheng, J. L. Sonnenberg, M. Hada, M. Ehara, K. Toyota, R. Fukuda, J. Hasegawa, M. Ishida, T. Nakajima, Y. Honda, O. Kitao, H. Nakai, T. Vreven, J. A. Montgomery, Jr., J. E. Peralta, F. Ogliaro, M. Bearpark, J. J. Heyd, E. Brothers, K. N. Kudin, V. N. Staroverov, R. Kobayashi, J. Normand, K. Raghavachari, A. Rendell, J. C. Burant, S. S. Iyengar, J. Tomasi, M. Cossi, N. Rega, J. M. Millam, M. Klene, J. E. Knox, J. B. Cross, V. Bakken, C. Adamo, J. Jaramillo, R. Gomperts, R. E. Stratmann, O. Yazyev, A. J. Austin, R. Cammi, C. Pomelli, J. W. Ochterski, R. L. Martin, K. Morokuma, V. G. Zakrzewski, G. A. Voth, P. Salvador, J. J. Dannenberg, S. Dapprich, A. D. Daniels, Ö. Farkas, J. B. Foresman, J. V. Ortiz, J. Cioslowski, and D. J. Fox, *Gaussian 09* (Gaussian, Inc., Wallingford CT, **2009**).

10 A.D. Becke *J. Chem. Phys.* **1993**, *98*, 5648.

11 A. D. Becke *J. Chem. Phys.* **1993**, *98*, 1372.

12 Y. Zhao, D. G. Truhlar *Theor. Chem. Acc.* **2008**, *120*, 215.

13 P. J. Hay and W. R. Wadt, *J. Chem. Phys.*, **1985**, *82*, 299.

14 a) G. A. Petersson, A. Bennett, T. G. Tensfeldt, M. A. Al-Laham, W. A. Shirley, and J. Mantzaris *J. Chem. Phys.*, **1988**, *89*, 2193; b) G. A. Petersson and M. A. Al-Laham *J. Chem. Phys.* **1991**, *94*, 6081.

15 a) V. Barone, M. Cossi *J. Phys. Chem. A*, **1998**, *102*, 1995; b) M. Cossi, N. Rega, G. Scalmani, V. Barone *J. Comp. Chem.*, **2003**, *24*, 669.

16 a) GaussView, Version 5, R. Dennington, T. Keith, and J. Millam, Semichem Inc., Shawnee Mission, KS, **2009**; b) https://www.chemcraftprog.com.
